# Rbbp4 loss disrupts neural progenitor cell cycle regulation independent of Rb and leads to Tp53 acetylation and apoptosis

**DOI:** 10.1002/dvdy.467

**Published:** 2022-03-18

**Authors:** Laura E. Schultz‐Rogers, Michelle L. Thayer, Sekhar Kambakam, Wesley A. Wierson, Jordan A. Helmer, Mark D. Wishman, Kristen A. Wall, Jessica L. Greig, Jaimie L. Forsman, Kavya Puchhalapalli, Siddharth Nair, Trevor J. Weiss, Jon M. Luiken, Patrick R. Blackburn, Stephen C. Ekker, Marcel Kool, Maura McGrail

**Affiliations:** ^1^ Department of Genetics, Development and Cell Biology Iowa State University Ames Iowa USA; ^2^ Interdepartmental Graduate Program in Genetics and Genomics Iowa State University Ames Iowa USA; ^3^ Interdepartmental Graduate Program in Molecular, Cellular and Developmental Biology Iowa State University Ames Iowa USA; ^4^ Genetics Iowa State University Ames Iowa USA; ^5^ Biology Iowa State University Ames Iowa USA; ^6^ Kinesiology and Health Iowa State University Ames USA; ^7^ Department of Biochemistry and Molecular Biology Mayo Clinic Rochester Minnesota USA; ^8^ Hopp Children's Cancer (KiTZ) Heidelberg Germany; ^9^ Division of Pediatric Neuro‐oncology, German Cancer Research Center (DKFZ), and German Cancer Consortium (DKTK) Heidelberg Germany; ^10^ Princess Maxima Center for Pediatric Oncology Utrecht Netherlands; ^11^ Present address: Department of Pathology and Lab Medicine University of North Carolina Chapel Hill North Carolina USA; ^12^ Present address: Department of Pathology St. Jude Children's Research Hospital Memphis Tennessee USA

**Keywords:** cell cycle regulation, embryo, neural precursor, neurogenesis, Tp53

## Abstract

**Background:**

Retinoblastoma binding protein 4 (Rbbp4) is a component of transcription regulatory complexes that control cell cycle gene expression. Previous work indicated that Rbbp4 cooperates with the Rb tumor suppressor to block cell cycle entry. Here, we use genetic analysis to examine the interactions of Rbbp4, Rb, and Tp53 in zebrafish neural progenitor cell cycle regulation and survival.

**Results:**

Rbbp4 is upregulated across the spectrum of human embryonal and glial brain cancers. Transgenic rescue of *rbbp4* mutant embryos shows Rbbp4 is essential for zebrafish neurogenesis. Rbbp4 loss leads to apoptosis and γ‐H2AX in the developing brain that is suppressed by *tp53* knockdown or maternal zygotic deletion. Mutant retinal neural precursors accumulate in M phase and fail to initiate G0 gene expression. *rbbp4*; *rb1* mutants show an additive effect on the number of M phase cells. In *rbbp4* mutants, Tp53 acetylation is detected; however, Rbbp4 overexpression did not rescue DNA damage‐induced apoptosis.

**Conclusion:**

Rbbp4 is necessary for neural progenitor cell cycle progression and initiation of G0 independent of Rb. Tp53‐dependent apoptosis in the absence of Rbpb4 correlates with Tp53 acetylation. Together these results suggest that Rbbp4 is required for cell cycle exit and contributes to neural progenitor survival through the regulation of Tp53 acetylation.

## Key Findings

- Rbbp4 regulates neural progenitor cell cycle independent of Rb
- Rbbp4 loss leads to Tp53 acetylation, gamma‐H2AX labeling and apoptosis in neural progenitors
- Suppression of gamma‐H2AX labeling and apoptosis by Tp53 knockdown in Rbbp4 mutants indicates Rbpp4 may induce apoptosis by transcriptional activation of Tp53 independent of the DNA damage response pathway

## 1 INTRODUCTION

The WD‐repeat protein retinoblastoma binding protein 4 (Rbbp4) functions as a chromatin assembly factor and adaptor for multiple chromatin remodelers and transcriptional regulators.^1^, ^2^, ^3^ These include the H3K27 methyltransferase polycomb repressive complex PRC2^2^ and the histone acetyltransferase activator p300/CREB.^4^ Rbbp4 is also a member of protein complexes that play critical roles in the regulation of cell cycle gene expression. Rbbp4 was first discovered as a binding partner of the tumor suppressor Rb in yeast and shown to be required for the suppression of Ras‐mediated growth.^5^ The nucleosome remodeling and histone deacetylase (HDAC) complex NuRD contains Rbbp4 and its closely related homolog Rbbp7.^6^, ^7^, ^8^ NuRD is recruited to E2F promoters by the Rb tumor suppressor and represses early S gene transcription.^9^ Early studies in *Caenorhabditis elegans* identified the Rbbp4 homolog *lin‐53* as a component of the MuvB complex, which negatively regulates vulval precursor cell fate by *lin‐35* (Rb‐like) repression of genes required for vulval induction.^10^ In *Drosophila* and humans, MuvB functions as a core that associates with Rb‐like and B‐Myb transcription factors to form the multisubunit complexes DREAM/Rb‐like and MMB/B‐Myb.^11^ DREAM and MBB complexes regulate stage‐specific gene expression during cell cycle progression^12^ and quiescence.^13^ Together these studies indicate that Rbbp4 functions in regulation of cell cycle progression and cell fate specification through transcriptional regulation by Rb family members and NuRD, DREAM, and MMB.

Rb has been shown to be essential for embryonic and adult neurogenesis in mice.^14^, ^15^ Our previous genetic analyses of Rb and Rbbp4 mutants in zebrafish demonstrate both are required for neurogenesis during brain development.^16^, ^17^ Live brain imaging revealed that zebrafish *rb1* mutant neural progenitors enter the cell cycle and fail to progress through mitosis, whereas the loss of *rbbp4* leads to neural progenitor apoptosis.^17^ While in yeast and cultured cells, Rbbp4 appears to function as a tumor suppressor with Rb to block cell cycle entry and inhibit cell growth, whether Rb and Rbbp4 interact to regulate the cell cycle in neural progenitors has not previously been examined. In both zebrafish Rb germline mutants and Rb‐defective brain tumors, Rbbp4 is overexpressed,^17^ suggesting Rbbp4 adopts oncogenic properties in the absence of Rb. In humans, Rbbp4‐dependent recruitment of chromatin regulators has been demonstrated in glioblastoma multiforme cell resistance to temozolomide through p300 activation of DNA repair pathway genes.^18^ Rbbp4 was also shown to be required for tumor progression in neuroblastoma xenografts by PRC2 silencing of tumor suppressors.^19^ Given the important contributions of developmental and cell cycle control mechanisms in cancer,^12^, ^20^ a more detailed examination of the role of Rbbp4 in neural progenitor cell cycle regulation would provide insight into its possible oncogenic roles in brain tumors.

A previous study using mouse primary cortical neurons demonstrated that chemical inhibition of HDAC led to increased TP53 acetylation, which protected cells from DNA damage induced apoptosis by preventing TP53 association with the promoter of the pro‐apoptotic gene PUMA.^21^ In contrast, both NuRD^22^ and Sirt1^23^, ^24^ histone deacetylase 1 complexes have been shown to deacetylate TP53 and repress TP53 transcriptional activation of apoptosis in human and mouse cell lines. Although these in vitro studies suggest opposite effects of Tp53 acetylation on apoptosis, together they indicate TP53 acetylation has a critical role in the cellular transcriptional response to stress. In vivo studies examining whether Tp53 acetylation is associated with transcriptional activation and induction of apoptosis may resolve these differences.

In this study, we use *rbbp4*, *rb1*, and *tp53* zebrafish mutants to further investigate the in vivo role of Rbbp4 in neurogenesis and examine the interaction of Rbbp4, Rb, and Tp53 in neural progenitor cell cycle regulation and survival. *rbbp4* mutant neural progenitors accumulate in M‐phase and Tp53‐dependent apoptosis occurs throughout the developing midbrain and retina. In situ analysis demonstrates that *rbbp4* mutant progenitors are lost before initiating G0 *cdkn1c* cyclin‐dependent kinase inhibitor gene expression and exiting the cell cycle. In double mutant *rbbp4*; *rb1* midbrain and retina apoptosis still occurs, and the level of M‐phase cells is additive, indicating independent functions for Rbbp4 and Rb in neural progenitor cell cycle regulation. In the absence of Tp53, apoptosis and γ‐H2AX labeling is suppressed in *rbbp4* mutant neural precursors, leading to increased accumulation of cells in M phase of the cell cycle. In contrast to wild‐type, Tp53 acetylation can be detected in homozygous mutant *rbbp4* embryos. Together these results suggest one mechanism by which Rbbp4 controls neural progenitor survival and cell cycle progression is through inhibition of Tp53 acetylation, providing new insight into an oncogenic role for Rbbp4 in brain cancer.

## 2 RESULTS

### 2.1 Human *RBBP4* is upregulated in pediatric embryonal and adult malignant brain cancers

**FIGURE 1:**
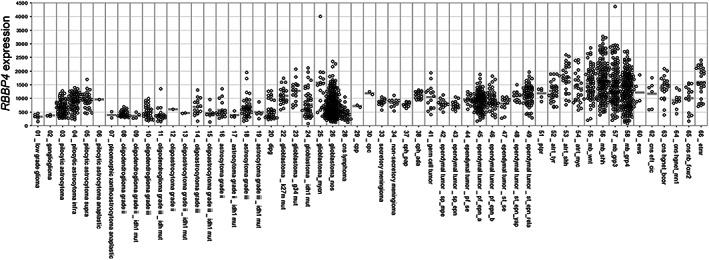
*RBBP4* is highly expressed in many malignant pediatric and adult brain cancers. *RBBP4* Affymetrix data from 2284 human tumor samples (German Cancer Research Center DKFZ). Y axis represents MAS5.0 normalized expression values. atrt, atypical teratoid/rhabdoid tumor; cns, central nervous system; cpc, choroid plexus carcinoma; cph, choroid plexus hyperplasia; cpp, choroid plexus papilloma; dipg, diffuse intrinsic pontine glioma; etmr, embryonal tumor with multilayered rosettes; ews, Ewing's sarcoma; mb, medulloblastoma; ptpr, papillary tumor of the pineal

We previously showed by RNA‐Seq and qRT‐PCR that *rbbp4* is upregulated ~8‐fold in a zebrafish model of *rb1*‐defective brain tumors^17^ that are similar to human primitive neuroectodermal tumors, a poorly differentiated and highly malignant pediatric brain cancer. To investigate whether human *RBBP4* is upregulated in human embryonal tumors and other aggressive pediatric and adult brain cancers we examined *RBBP4* expression data from 2284 human brain tumor samples from the German Cancer Research Center. Elevated *RBBP4* expression was detected in tissues from many embryonal central nervous system primitive neuroectodermal tumors (CNS‐PNETs) and malignant brain cancers, including ependymal, glial, oligodendroglial, and astrocytic tumors (Figure 1). We previously used CRISPR/Cas9 gene editing to isolate a 4 bp deletion allele in zebrafish *rbbp4* ^Δ4*is*60^ and showed *rbbp4* ^Δ4^ is homozygous mutant larval lethal.^17^ Loss of Rbbp4 leads to apoptosis throughout the 2 day post‐fertilization (dpf) brain and retina, resulting in microcephaly and microphthalmia.^17^ Together these results show that elevated *RBBP4* is a common signature in aggressive central nervous system tumors and suggest that Rbbp4 may contribute to oncogenesis by promoting brain tumor cell survival.

### 2.2 Rbbp4 is essential for zebrafish neurogenesis

**FIGURE 2:**
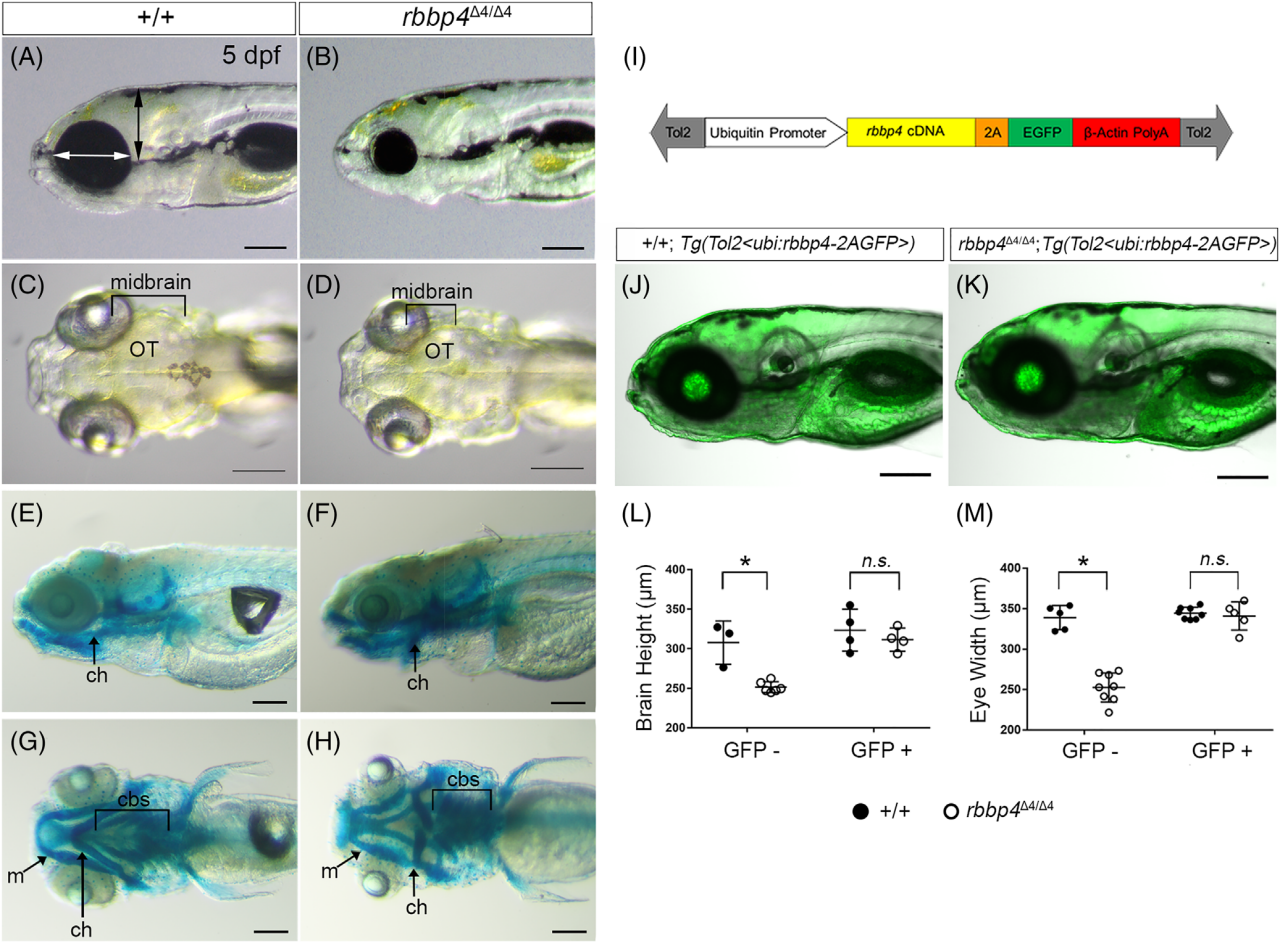
Rbbp4 is essential for zebrafish brain and neural crest development and persistent neurogenesis in the midbrain optic tectum and retina. (A) Lateral view of 5 dpf wild‐type zebrafish indicating location of measurements for head height (black arrow) and eye width (white arrow). (B) Lateral view of 5 dpf *rbbp4* ^Δ4/Δ4^ homozygote showing gross defects including microcephaly and microphthalmia. (C, D) Dorsal views of wild type and *rbbp4* ^Δ4/Δ4^ homozygous larvae show the reduced size of the midbrain optic tectum in the *rbbp4* mutant. Lateral and ventral views of 5 dpf wild type (E, G) and *rbbp4* ^Δ4/Δ4^ homozygous (F, H) larvae stained with alcian blue to reveal cartilage structures. (I–J) *rbbp4* cDNA rescue of *rbbp4* ^Δ4/Δ4^ homozygotes. (I) Schematic of *Tol2*<*ubi:rbbp4‐2AGFP*> cDNA rescue construct driving expression of *rbbp4‐2AGFP* from the *ubiquitin* promoter. (J) 5 dpf transgenic +/+; *Tg*(*Tol2*<*ubi:rbbp4‐2AGFP*>) and (K) 5 dpf transgenic *rbbp4* ^Δ4/Δ4^; *Tg*(*Tol2*<*ubi:rbbp4‐2AGFP*>) larvae. (L) *rbbp4* ^Δ4/Δ4^ homozygotes (n = 6) have a significantly smaller head than wild type (n = 3, *P* = .0015). Transgenic GFP+ *rbbp4* ^Δ4/Δ4^; *Tg*(*Tol2*<*ubi:rbbp4‐2AGFP*>) homozygotes (n = 4) show no significant difference in head height compared to +/+; *Tg*(*Tol2*<*ubi:rbbp4‐2AGFP*>) (n = 4) (*P* = .4595). (M) *rbbp4* ^Δ4/Δ4^ homozygotes (n = 8) have a significantly smaller eye than wild type (n = 5, *P* = .0001). Transgenic *rbbp4* ^Δ4/Δ4^; *Tg*(*Tol2*<*ubi:rbbp4‐2AGFP*>) GFP+ (n = 5) show no significant difference in eye size compared to +/+; *Tg*(*Tol2* < *ubi*:*rbbp4‐2AGFP*>) (n = 8, *P* = .6293). Data represent mean ± s.e.m. *P* values calculated with two‐tailed Student's *t*‐test. cbs, ceratobranchial cartilage; ch, ceratohyal cartilage; m, Meckel's cartilage; OT, optic tectum. Data confirming individual genotypes are presented in Table 1. Scale bars 100 μm

To demonstrate Rbbp4 is essential for cell survival during neurogenesis, we further characterized the zebrafish *rbbp4* ^Δ4^ mutant and its impact on brain development. We tested for rescue of the homozygous *rbbp4* ^Δ4^ mutant using a transgenic wild‐type *rbbp4* cDNA. Homozygous mutant *rbbp4* ^Δ4/Δ4^ larvae display severe defects in neurogenesis, presenting with microcephaly and microphthalmia^17^ (Figure 2A–D). Alcian blue staining revealed abnormalities in formation of head cartilage structures (Figure 2E–H), including the ceratohyal cartilage (ch), ceratobranchial cartilage (cbs), and Meckel's cartilage (m). These results suggest that Rbbp4 is necessary for the development of the central nervous system. Ubiquitous overexpression of *rbbp4* cDNA by a *Tol2 < ubi*:*rbbp4‐2AGFP >* transgenic line (Figure 2I) was tested for the ability to rescue the *rbbp4* ^Δ4/Δ4^ phenotype. The transgene did not affect development in wild‐type embryos or viability and fertility in adults (Figure 2J, Table 1) and was able to rescue the gross morphological defects in *rbbp4* ^Δ4/Δ4^ mutants (Figure 2K). Midbrain height and eye width measurements in *Tol2<ubi*:*rbbp4‐2AGFP>* and *rbbp4* ^Δ4/Δ4^; *Tol2<ubi*:*rbbp4‐2AGFP>* transgenic embryos confirmed rescued animals showed no significant difference in morphology from wild type (Figure 2L,M). *rbbp4* ^Δ4/Δ4^; *Tg*(*Tol2<ubi*:*rbbp4‐2AGFP>*) individuals are viable and become fertile adults. Rescue of the *rbbp4* ^Δ4/Δ4^ phenotype by the *Tg*(*Tol2<ubi*:*rbbp4‐2AGFP>*) transgene demonstrates Rbbp4 is essential for larval viability, midbrain and retina development. While the defect in head cartilage suggests that *rbbp4* may be required for neural crest‐derived structures, it is possible that the effect is indirectly caused by a disruption of mesoderm or endodermal pouch formation.

**TABLE 1:** Frequency of progeny genotypes and phenotypes from heterozygous *rbbp* ^Δ4^ */+* crossed to heterozygous *rbbp* ^Δ4^/*+*; *Tg*(*Tol2<ubi:rbbp4‐2AGFP>)* zebrafish

	Total number of progeny	GFP negative						GFP positive					
Cross		Total GFP‐	Normal Phenotype	Mutant Phenotype	*rbbp4* +/+	*rbbp4*△4*/+*	*rbbp4*△4*/*△4	Total GFP+	Normal phenotype	Mutant phenotype	rbbp4 +/+	*rbbp4*△4*/+*	*rbbp4*△4*/*△*4*
Clutch 1	76	39/76 (51.3%)	28/39 (71.8%)	11/39 (28.2%)	n/a	n/a	n/a	37/76 (48.7%)	37/37 (100%)	0/37 (0%)	8/37 (21.6%)	17/37 (45.9%)	12/37 (32.4%)
Clutch 2	108	61/108 (56.4%)	48/61 (78.6%)	13/61 (21.3%)	5/24 (20.8%)	11/24 (45.8%)	8/24 (33.3%)	47/108 (43.5%)	47/47 (100%)	0/47 (0%)	8/24 (33.3%)	11/24 (45.8%)	5/24 (20.8%)
Clutch *3*	307	155/307 (50.5%)	110/155 (71%)	45/155 (29%)	18/48 (47.3%)	17/48 (35.4%)	13/48 (27%)	152/307 (49.5%)	152/152 (100%)	0/152 (0%)	16/48 (33.3%)	22/48 (45.8%)	10/48 (20.8%)

*Note*: Three independent crosses were set up between female *rbbp4* ^Δ4^ */+* heterozygotes and male *rbbp4* ^Δ4^ */+*; *Tg(Tol2<ubi*:*rbbp4‐2AGFP>)* transgenic heterozygotes. Embryo clutches were sorted into positive and negative GFP groups at 2 dpf. At 5 dpf larvae were sorted by phenotype and then genotyped. “Total” column shows approximately 50% GFP− and GFP+ in each clutch, indicating the *Tg*(*Tol2<ubi*:*rbbp4‐2AGFP>*) line contains a single transgene integration. Scoring of GFP negative embryos revealed close to Mendelian segregation of the *rbbp4* ^Δ4/Δ4^ gross mutant phenotype in one quarter of the progeny (28.2%, 21.3%, 29%). A random sampling of embryos from clutches 2 and 3 was genotyped and confirmed Mendelian segregation of the phenotype with the *rbbp4* ^Δ4^ allele. In each clutch, none of the GFP positive embryos showed the rbbp4 mutant phenotype. Random sampling and genotyping confirmed ~ one quarter of morphologically normal GFP+ larvae were homozygous mutant *rbbp4* ^Δ4/Δ4^ (32.4%, 20.8%, 20.8%).

### 2.3 Rbbp4 is required for cell cycle regulation and its loss leads to neural precursor apoptosis

**FIGURE 3:**
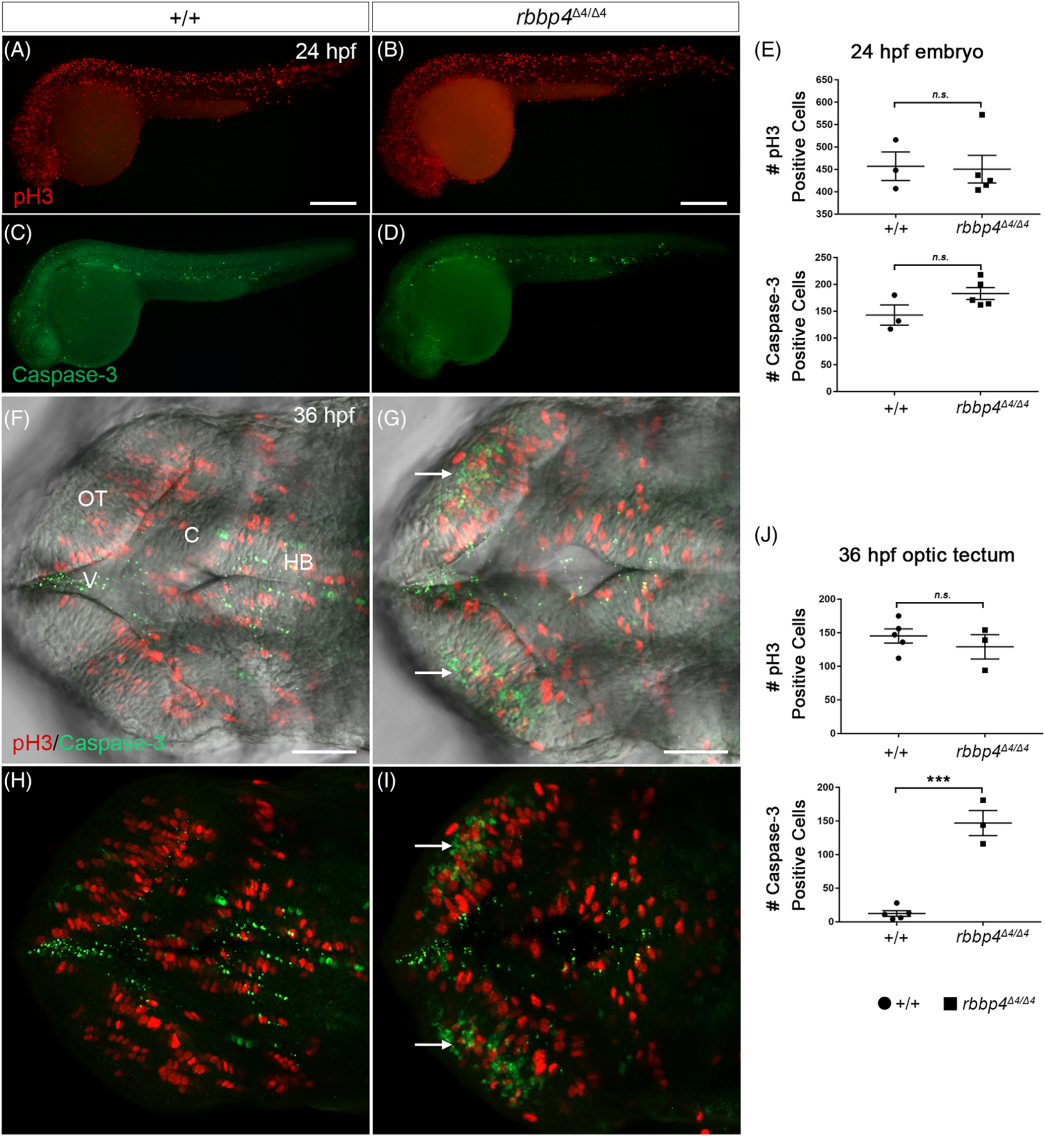
Apoptosis of *rbbp4* mutant neural progenitors can be detected in the 36 hpf embryonic brain optic tectum. pH 3 and activated Caspase‐3 labeling in 24 hpf (A–E) and 36 hpf (F–J) wild type and *rbbp4*Δ4/Δ4 mutant embryos. At 24 hpf the levels of pH 3 (A, B) and activated Caspase‐3 (C, D) throughout the embryo are not significantly different (E); 24 hpf wild‐type (n = 3) and homozygous *rbbp4*Δ4/Δ4 (n = 5), pH 3, *P* = .8966 ns; activated Caspase‐3 *P* = .0960 ns. (F–I) Dorsal views of the optic tectum in 36 hpf wild type (F, H) and *rbbp4*Δ4/Δ4 (G, I) mutant embryos shows significantly elevated levels of activated Caspase‐3 while the number of pH 3 mitotic cells is not significantly different (J). The 36 hpf wild type (n = 3) and heterozygous *rbbp4*Δ4/+ (n = 3), activated Caspase‐3 *P* = .0001***; pH 3 *P* = .4310 n.s. Statistical analysis was performed with an unpaired, two tailed Student's *t*‐test. Plots show mean ± s.e.m. C, cerebellum; HB, hindbrain; n.s., not significant; OT, optic tectum. Scale bars: A, B 150 μm; F, G 50 μm

We previously showed that apoptosis is present throughout the midbrain and retina in 2 dpf homozygous mutant *rbbp4* ^Δ4/Δ4^ larvae,^17^ which suggested that Rbbp4 is required for survival of neural precursors. To determine whether the loss of Rbbp4 affects neural precursor proliferation or survival earlier in embryogenesis we examined phosphohistone H3 and activated Caspase‐3 levels at 24 and 36 hours post fertilization (hpf). At 24 hpf (Figure 3A–E), whole mount labeled +/+ wild type (Figure 3A,C) and *rbbp4* ^Δ4/Δ4^ mutant (Figure 3B,D) embryos did not show a significant difference in the number of pH 3 (*P* = .8966) or activated Caspase‐3 (*P* = .0960) positive cells (Figure 3E). Like wild‐type embryos, at 36 hpf, *rbbp4* ^Δ4/Δ4^ mutant embryos appear morphologically normal and have well defined brain structures, including the optic tectum, cerebellum, and hindbrain (Figure 3F,G). Confocal microscopy was used to image pH 3 and activated Caspase‐3 in the dorsal optic tectum of whole mount‐labeled embryos (Figure 3F–J). The levels of pH 3 positive cells were not significantly different between 36 hpf +/+ wild type and *rbbp4* ^Δ4/Δ4^ mutant embryos (Figure 3J; *P* = .4310). However, compared to +/+ wild type (Figure 3F,H), in the *rbbp4* ^Δ4/Δ4^ mutant embryos showed significantly elevated levels of activated Caspase‐3 in the optic tectum (Figure 3G,H arrows; J; *P* = .0001). These results indicate that during embryonic neurogenesis, the loss of Rbbp4 did not impact proliferation of neuroepithelial cells but was required to prevent the apoptosis in the optic tectum.

**FIGURE 4:**
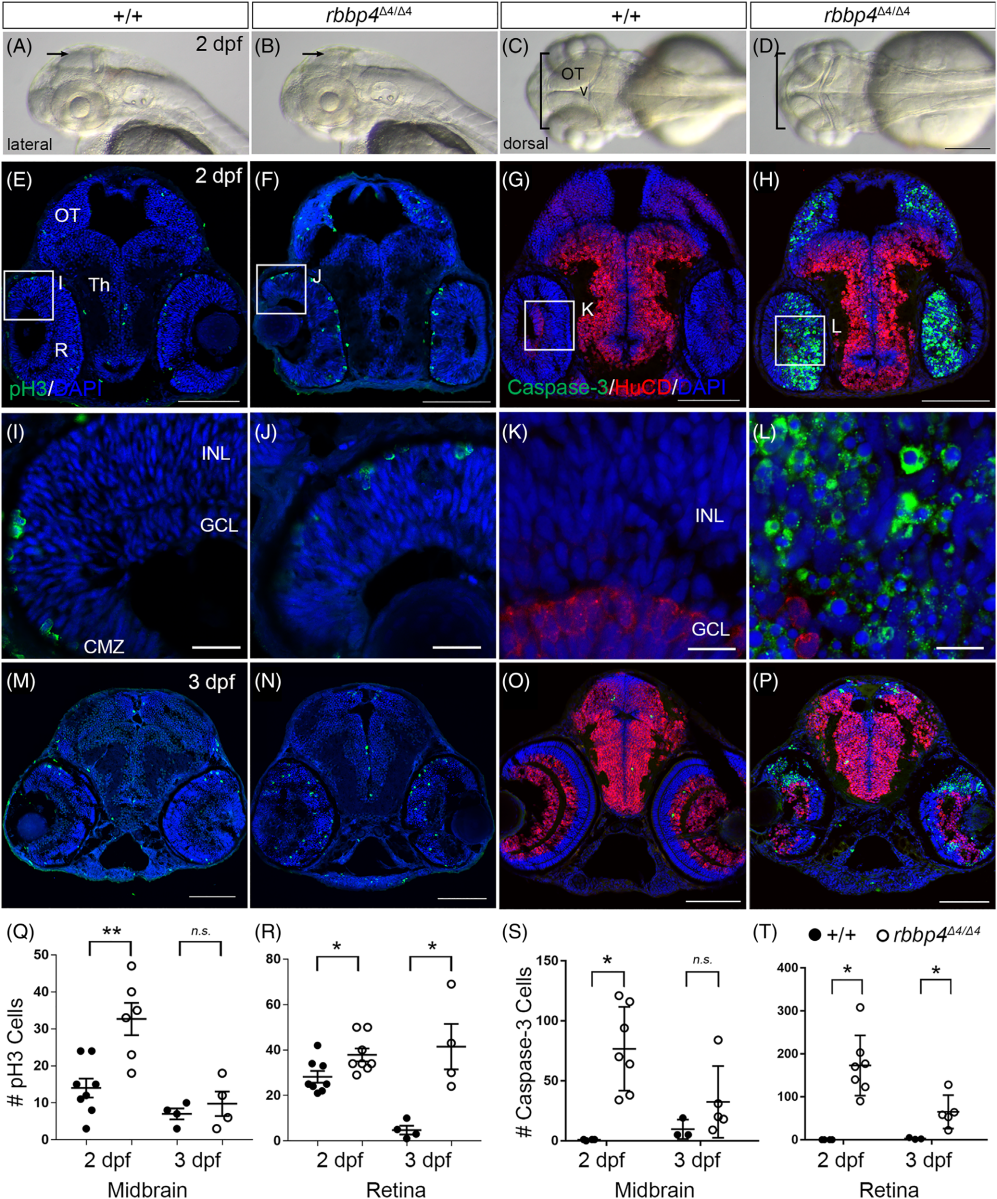
Increased levels of mitotic phase cells and apoptotic cells are present in *rbbp4* ^Δ4/Δ4^ mutant larval midbrain and retina. Lateral (A, B) and dorsal (C, D) images of 2 dpf larva show the size of the optic tectum (arrows, OT) and midbrain (brackets) is visibly reduced in *rbbp4* ^Δ4/Δ4^ mutants compared to +/+ wild type. (E–P) Transverse sections of zebrafish midbrain and retina labeled with DAPI and antibodies to the mitotic marker pH 3 (green), or apoptosis marker activated Caspase‐3 (green) and neural marker HuC/D (red). (E, F, I, J) 2 dpf and (M, N) 3 dpf pH 3 labeled tissue. (I, J) Higher magnification of retina from boxed area in E, F. (G, H, K, L) 2 dpf and (O, P) 3 dpf activated Caspase‐3 and HuC/D‐labeled tissue. (K, L) Higher magnification of retina from boxed area in G, H. (Q, R) Quantification of pH 3 positive cells in the midbrain (2 dpf *P* = .0058**; 3 dpf *P* = .4904 n.s.) and retina (2 dpf *P* = .0258*; 3 dpf *P* = .0324*). (S, T) Quantification of Caspase‐3 positive cells in the midbrain (2 dpf *P* = .0022*; 3 dpf *P* = .2569 n.s.) and retina (2 dpf *P* = .0010*; 3 dpf *P* = .0358*). Statistical analysis was performed with an unpaired, two tailed Welch's *t*‐test. Plots show mean ± s.e.m. CMZ, ciliary marginal zone; GCL, ganglion cell layer; INL, inner nuclear layer; n.s., not significant; OT, optic tectum; Th, thalamic region; R, retina. Scale bars: D 200 μm; E–H 100 μm; I, J 20 μm; K, L 10 μm; M–P 200 μm

The effect of loss of Rbbp4 on brain morphology was first evident in 2 dpf embryos (Figure 4A–D). Compared to wild type (Figure 4A,C, in *rbbp4* ^Δ4/Δ4^ (Figure 4B,D) the size of the optic tectum and midbrain–hindbrain structures appeared reduced (Figure 4A,B arrows; C,D brackets). Labeling of 2 and 3 dpf sectioned head tissue with pH 3 or activated Caspase‐3 (Figure 4E–P) revealed differences in both proliferation and apoptosis. pH 3 positive cells can be detected in the midbrain and retina in wild type (Figure 4E,I,M) and *rbbp4* ^Δ4/Δ4^ mutants (Figure 4F,J,N). The number of pH 3 positive cells at 2 dpf was significantly elevated in both *rbbp4* ^Δ4/Δ4^ mutant midbrain (Q *P* = .0058) and retina (R 0.0258). At 3 dpf, there was no difference in the midbrain (Q *P* = .4904), but the level of pH 3 cells in the retina remained significantly higher in the *rbbp4* ^Δ4/Δ4^ mutants (R *P* = .0324). At 2 and 3 dpf in wild type (Figure 4G,K,O), there are very few activated Caspase‐3 positive cells in the midbrain or retina. In contrast, as we reported previously, at 2 dpf activated Caspase‐3 labeling was significantly increased in *rbbp4* ^Δ4/Δ4^ mutants (Figure 4H,L,P) in both the midbrain (Figure 4S; *P* = .0022) and retina (Figure 4T; *P* = .0010). Activated Caspase‐3 labeling and fragmented nuclei were detected throughout the inner nuclear layer of the retina (Figure 4L). HuC/D labeling in the midbrain and retinal ganglion cell layer indicated that earlier born neurons were able to differentiate and survive in the absence of Rbbp4, possibly due to maternal contribution of Rbbp4. By 3 dpf, the number of activated Caspase‐3 positive cells in *rbbp4* ^Δ4/Δ4^ mutant midbrain tissue varied but was not significantly different than wild type (Figure 4S; *P* = .2569) and became restricted to the proliferative zones in the dorsal tectum. In the 3 dpf *rbbp4* ^Δ4/Δ4^ mutant retina, the level of activated Caspase‐3 positive cells remained significantly high (Figure 4T; *P* = .0358). Together, these results suggest that the loss of Rbbp4 leads to a failure in neural precursor cell cycle progression and induction of apoptosis in the midbrain and retina.

**FIGURE 5:**
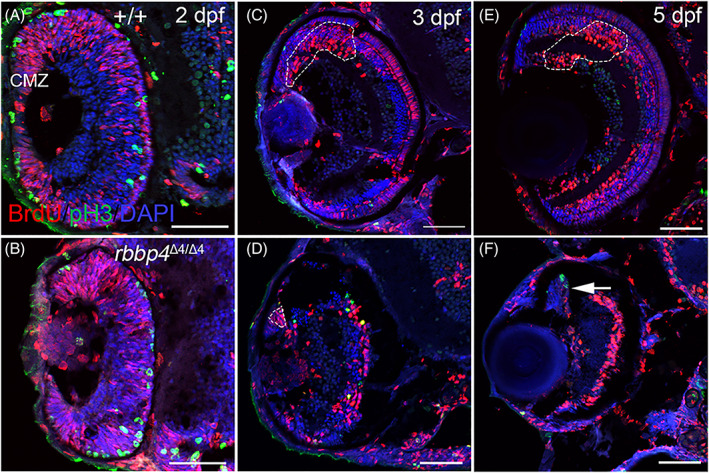
Ciliary marginal zone neural stem cells continue to divide but neural precursors fail to survive in *rbbp4* ^Δ4/Δ4^ homozygous mutant retina. (A–F) BrdU pulse‐chase labeling to examine the fate of newborn neurons in the *rbbp4* ^Δ4/Δ4^ mutant retina. (A, B) 2 dpf zebrafish embryos treated with a 2.5 hour BrdU pulse and immediately sacrificed. Transverse sections of retina from wild type (n = 4) and *rbbp4* ^Δ4/Δ4^ mutants (n = 5) were labeled with antibodies to BrdU and mitotic marker phospho‐Histone H3 (pH 3). BrdU labels proliferating cells at the retina ciliary marginal zone and cells scattered throughout the laminating retina. (C, D) Pulse‐chased larvae at 3 dpf. In wild‐type retina (n = 8), BrdU‐labeled cells are located in the region adjacent to the cmz where neural precursors and newly differentiated neurons reside (C outline). A small section of BrdU‐labeled cells remains at the ciliary marginal zone in the *rbbp4* ^Δ4/Δ4^ mutant retina (n = 6) (D outline). (E, F) Pulse‐chased larvae at 5 dpf. In wild type (n = 7), the BrdU‐labeled cells are now more centrally located in an older region of the growing retina (E outline). In the *rbbp4* ^Δ4/Δ4^ mutant retina (n = 7) mature retinal tissue is absent and BrdU‐negative stem cells persist at the ciliary marginal zone (F arrow). CMZ, ciliary marginal zone. Scale bars 50 μm

To examine which retinal cell populations fail to survive after loss of Rbbp4, BrdU pulse‐chase labeling was used to follow the fate of *rbbp4* ^Δ4/Δ4^ neural precursors in the post‐embryonic retina. Retinal neurogenesis proceeds in a conveyor belt pattern, with stem cells at the ciliary marginal zone generating progenitors that become progressively more committed as they move inward in the growing retina.^25^, ^26^ The 2 dpf embryos were exposed to a 3‐hour BrdU pulse and collected immediately or chased until 3 and 5 dpf. At the end of 2 dpf in wild‐type retina, BrdU is detected in stem cells at the retina ciliary marginal zone (CMZ) and progenitors in the inner nuclear and newly developing photoreceptor layers (Figure 5A). A similar pattern of BrdU incorporation was detected in the *rbbp4* ^Δ4/Δ4^ mutant retina (Figure 5B). At 3 dpf, BrdU‐labeled cells were found in the region adjacent to the CMZ in both wild type and *rbbp4* ^Δ4/Δ4^ mutants (Figure 5C,D outlines); however, many fewer BrdU positive cells were present in the mutant and the retina tissue was severely reduced. By 5 dpf, in wild‐type retina, the CMZ was devoid of BrdU positive cells, and BrdU positive cells were incorporated into the ganglion and inner nuclear layers of the laminated region of the wild‐type retina (Figure 5E). In the *rbbp4* ^Δ4/Δ4^ mutant cells at the CMZ were present but were not labeled with BrdU (Figure 5F arrow). Together, these results show that *rbbp4* ^Δ4/Δ4^ mutant retinal stem cells at the ciliary marginal zone survive and continue to divide, but the neural progenitor and/or newborn neuron cell populations undergoes apoptosis.

### 2.4 *rbbp4* mutant retinal neural precursors are lost prior to initiation of quiescence and differentiation

**FIGURE 6:**
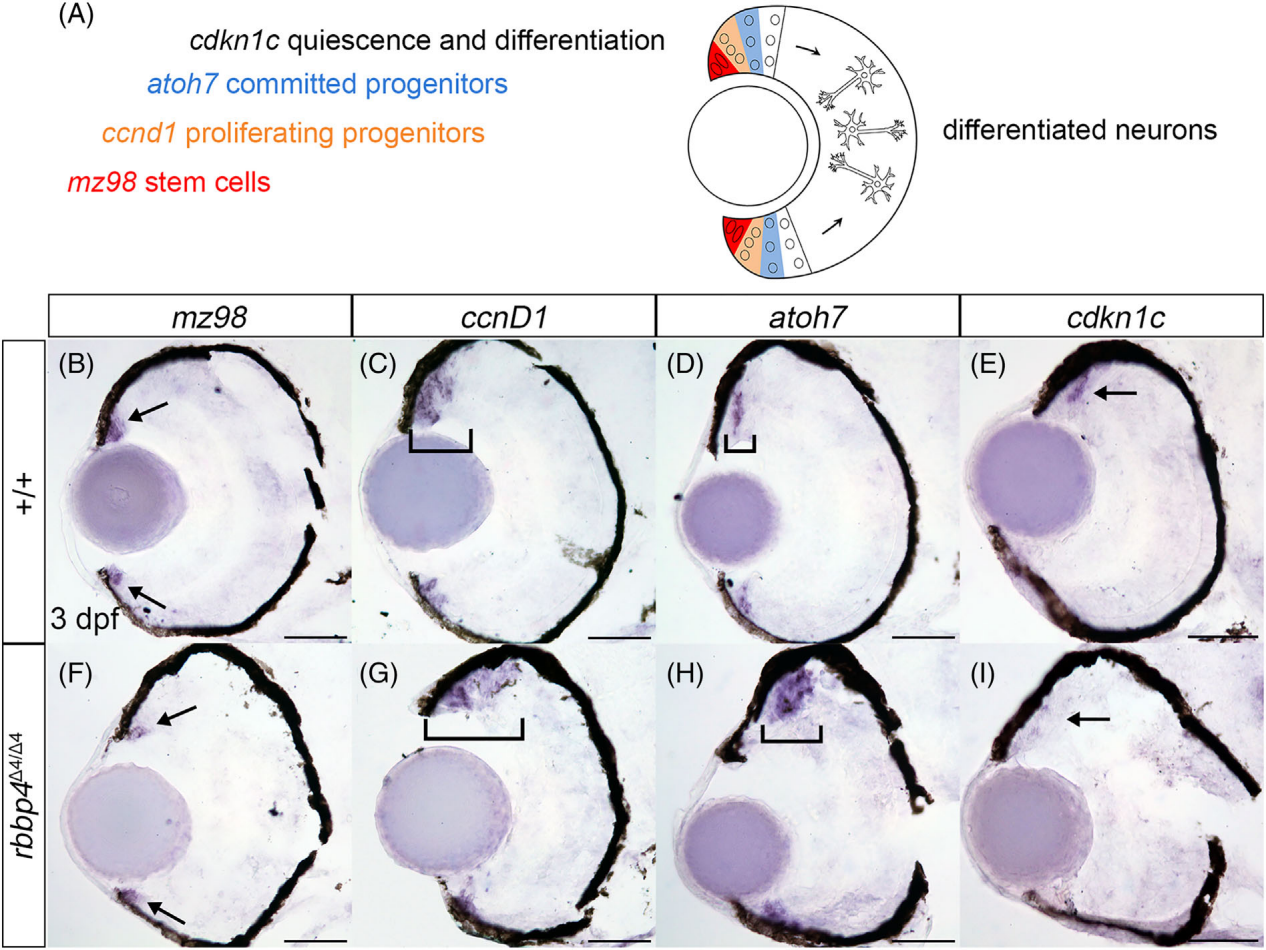
Neural precursors fail to initiate quiescence in the *rbbp4* ^Δ4/Δ4^ homozygous mutant retina. (A) Diagram of pattern of gene expression marking stages of neurogenesis in the developing zebrafish retina. (B–I) Transverse sections of 3 dpf zebrafish retina labeled by in situ hybridization to examine expression of the stem cell marker *mz98* (B, F arrows), proliferating progenitor marker *ccnD1* (C, G brackets), committed progenitor marker *atoh7* (D, H brackets) and differentiating precursor marker *cdkn1c* (E, I arrows). (B–E) Wild‐type retina (n = 5) shows location of progressively committed precursor cell populations. (F–I) In the *rbbp4* ^Δ4/Δ4^ homozygous mutant retina (n = 3) the proliferating and committed neural progenitor cell populations are expanded compared to wild type. *cdkn1c* expression is absent in the *rbbp4* ^Δ4/Δ4^ mutant retina (I arrow). Scale bars: 50 μm

Neurogenesis in the zebrafish larval retina can be visualized at 3 dpf by expression of genes in discreet sectors that mark progressive steps in neural progenitor commitment^27^ (Figure 6A). To identify the stage at which *rbbp4* ^Δ4/Δ4^ neural precursors are lost we performed in situ hybridization on 3 dpf wild type and *rbbp4* ^Δ4/Δ4^ retina with probes to collagen type XV alpha 1b (*mz98*),^28^ cyclin D1 (*ccnD1*),^29^ atonal 7 (*atoh7*)^30^ and G1 cyclin‐dependent kinase inhibitor 1Ca (*cdkn1c*)^31^ (Figure 6B–I). In wild type, cells at the periphery of the ciliary marginal zone express stem cell marker *mz98* (Figure B arrow), followed by proliferating progenitors that express *ccnD1* (Figure 6C bracket), and then *atoh7*‐expressing committed neural precursors (Figure 6D bracket). Lastly, *cdkn1c* expression marks precursors arrested in G1 that will initiate quiescence and differentiation (Figure 6E arrow). In the *rbbp4* ^Δ4/Δ4^ mutant retina, *mz98*‐expressing cells were present in the ciliary marginal zone (Figure 6F arrow). The population of *ccnd1‐*labeled progenitors and *atoh7*‐expressing committed precursors was significantly expanded in the mutant compared to wild type (Figure 6G,H brackets). However, *cdkn1c*‐expression was not detected in the next adjacent sector in the *rbbp4* ^Δ4/Δ4^ mutants (Figure 6I arrow). These results indicate that in the absence of Rbbp4, loss of committed retinal precursors adjacent to the ciliary marginal zone occurs before the cells transition to cell cycle exit and initiation of quiescence and differentiation. The expansion of the *ccnD1* and *atoh7* domains also suggests a failure in terminal differentiation.

### 2.5 Rbbp4 functions independently of Rb to regulate neural precursor cell cycle

**FIGURE 7:**
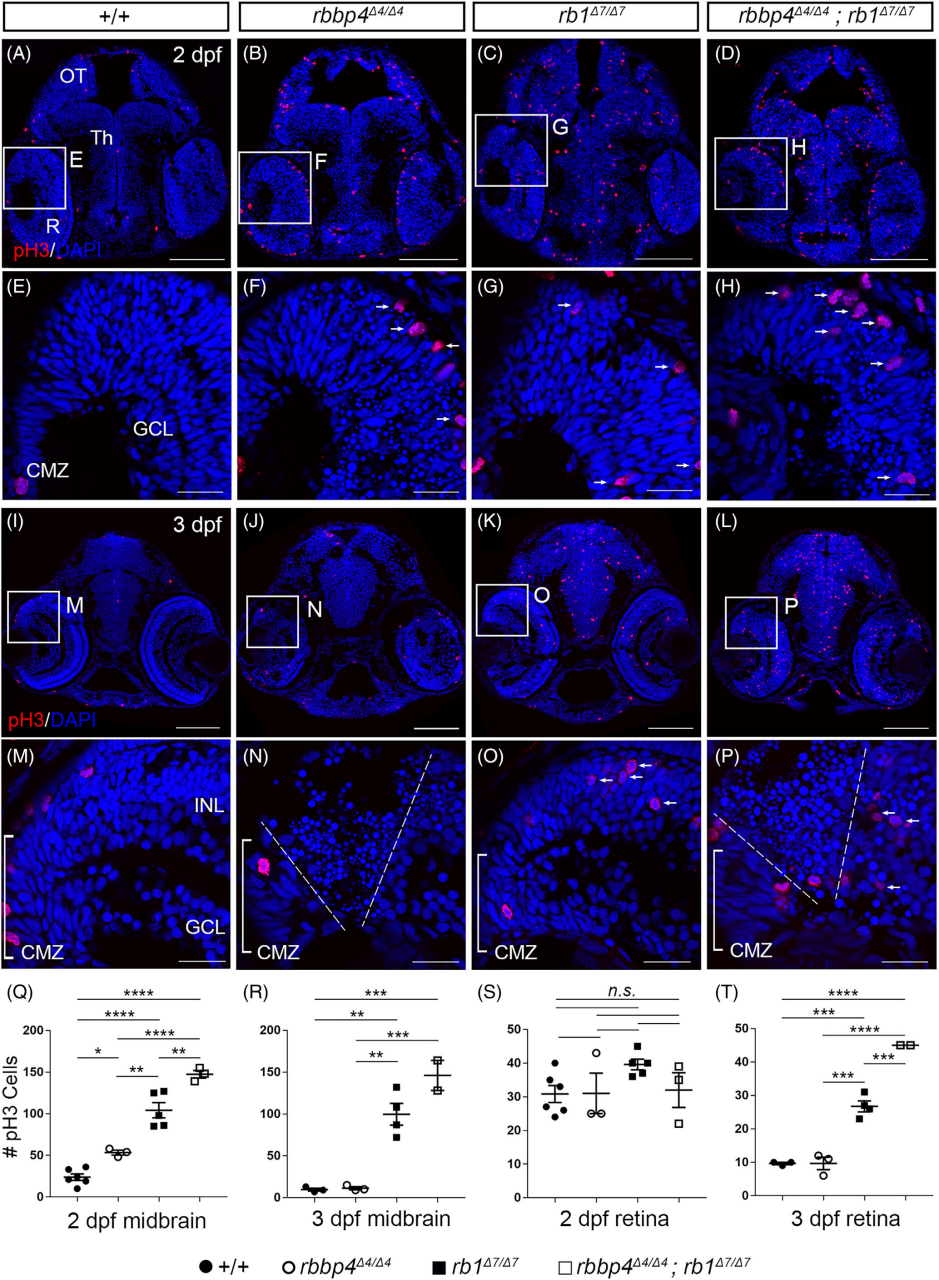
Loss of Rbbp4 and Rb have an additive effect on cell cycle regulation. (A–H) 2 dpf and (I–P) 3 dpf wild type +/+ (n = 6, n = 3), *rbbp4* ^Δ4/Δ4^ (n = 3, n = 3), *rb1* ^Δ7/Δ7^ (n = 5, n = 4), and *rbbp4* ^Δ4/Δ4^; *rb1* ^Δ7/Δ7^ (n = 3, n = 2) siblings from a *rbbp4* ^Δ4/+^; *rb1* ^Δ7/+^ incross were sectioned and labeled with antibodies to phosphohistone H3 (pH 3). Boxed area in A–D and I–L is shown at higher magnification in E‐H and M‐P, respectively. Small arrows point to pH 3‐positive mitotic cells. Dashed lines in N and P denote region adjacent to the ciliary marginal zone (brackets). (Q–T) Quantification of pH 3 positive cells. *P* values correspond to comparisons starting at highest line on each plot. Statistical analysis was performed with one‐way ANOVA Sidak's multiple comparisons test. Plots show mean ± s.e.m. (Q) 2 dpf midbrain (****P < .0001 ****P < . 0001, ****P < . 0001, *P = .0478, **P = .0042, **P = .0010). (R) 3 dpf midbrain (***P = .0002, **P = .0013, ***P = .0003, **P = .0014). (S) 2 dpf retina (*P* > .9999 n.s., *P* = .2855 n.s., *P* > .9999 n.s., *P* = .6331 n.s., *P* = .5042 n.s., *P* > .9999 n.s). (T) 3 dpf retina (****P < .0001, ***P = .0002, ****P < .0001, ***P = .0002, ***P = .0002, *P* > .9999 n.s.). CMZ, ciliary marginal zone; GCL, ganglion cell layer; INL, inner nuclear layer; ONL, outer nuclear layer; OT, optic tectum; R, retina; Th, thalamic region. Scale bars: A–D, I–L 100 μm; E–H, M–P 20 μm

We previously demonstrated that in *rb1* mutant zebrafish larvae neural progenitors can re‐enter the cell cycle and stall in M‐phase, using both live imaging of an H2A‐GFP reporter and pH 3 labeling.^17^ To determine whether Rbbp4 and Rb1 cooperate in regulating neural precursor cell cycle, we compared the levels of pH 3 positive mitotic phase cells in +/+, *rbbp4* ^Δ4/Δ4^, *rb1* ^Δ7/Δ7^ and *rbbp4* ^Δ4/Δ4^; *rb1* ^Δ7/Δ7^ double mutants at 2 dpf (Figure 7A–H) and 3dpf (Figure 7I–P). Figure 7 lists all significant *P* values. At 2 dpf in the midbrain *rbbp4* ^Δ4/Δ4^ (*P* = .0478), *rb1* ^Δ7/Δ7^ (*P* < .0001) and *rbbp4* ^Δ4/Δ4^; *rb1* ^Δ7/Δ7^ (*P* < .0001) contained significantly higher numbers of pH 3 positive cells in comparison to wild type (Figure 7Q). In the *rbbp4* ^Δ4/Δ4^; *rb1* ^Δ7/Δ7^ double mutants, the number of pH 3 positive cells was significantly greater than the *rbbp4* ^Δ4/Δ4^ (*P* < .0001) or *rb1* ^Δ7/Δ7^ (*P* = .0042) single mutants. At 3 dpf in the midbrain, the difference between wild type and *rbbp4* ^Δ4/Δ4^ was not significant (Figure 7R), nor was the difference between the *rb1* ^Δ7/Δ7^ single mutant and the *rbbp4* ^Δ4/Δ4^; *rb1* ^Δ7/Δ7^ double mutant. In the retina at 2 dpf the number of pH 3 positive cells (Figure 7F–Harrows) in *rbbp4* ^Δ4/Δ4^ (*P* > .9999), *rb1* ^Δ7/Δ7^ (*P* > .9999) or *rbbp4* ^Δ4/Δ4^; *rb1* ^Δ7/Δ7^ (*P* > .9999) was not significantly different from wild type (Figure 7S). However, in comparison to 3 dpf wild‐type retina, *rb1* ^Δ7/Δ7^ (*P* = .0002) and *rbbp4* ^Δ4/Δ4^; *rb1* ^Δ7/Δ7^ (*P* < .0001) had significantly higher levels pf pH 3 cells which were visible in the older part of the inner nuclear layer (Figure 7O,P arrows) adjacent to the sector where progenitors reside (Figure 7N,P dashed lines). The number of pH 3 positive cells in the 3 dpf double mutant *rbbp4* ^Δ4/Δ4^; *rb1* ^Δ7/Δ7^ retina was significantly greater than in *rb1* ^Δ7/Δ7^ mutant alone (*P* = .0002) (Figure 7T), but the level of increase indicates an additive effect. Together these results indicate *rbbp4* and *rb1* have independent functions in the regulation of neural progenitor cell cycle progression.

### 2.6 Rbbp4 is required for survival of *rb1* mutant retinal neural precursors

**FIGURE 8:**
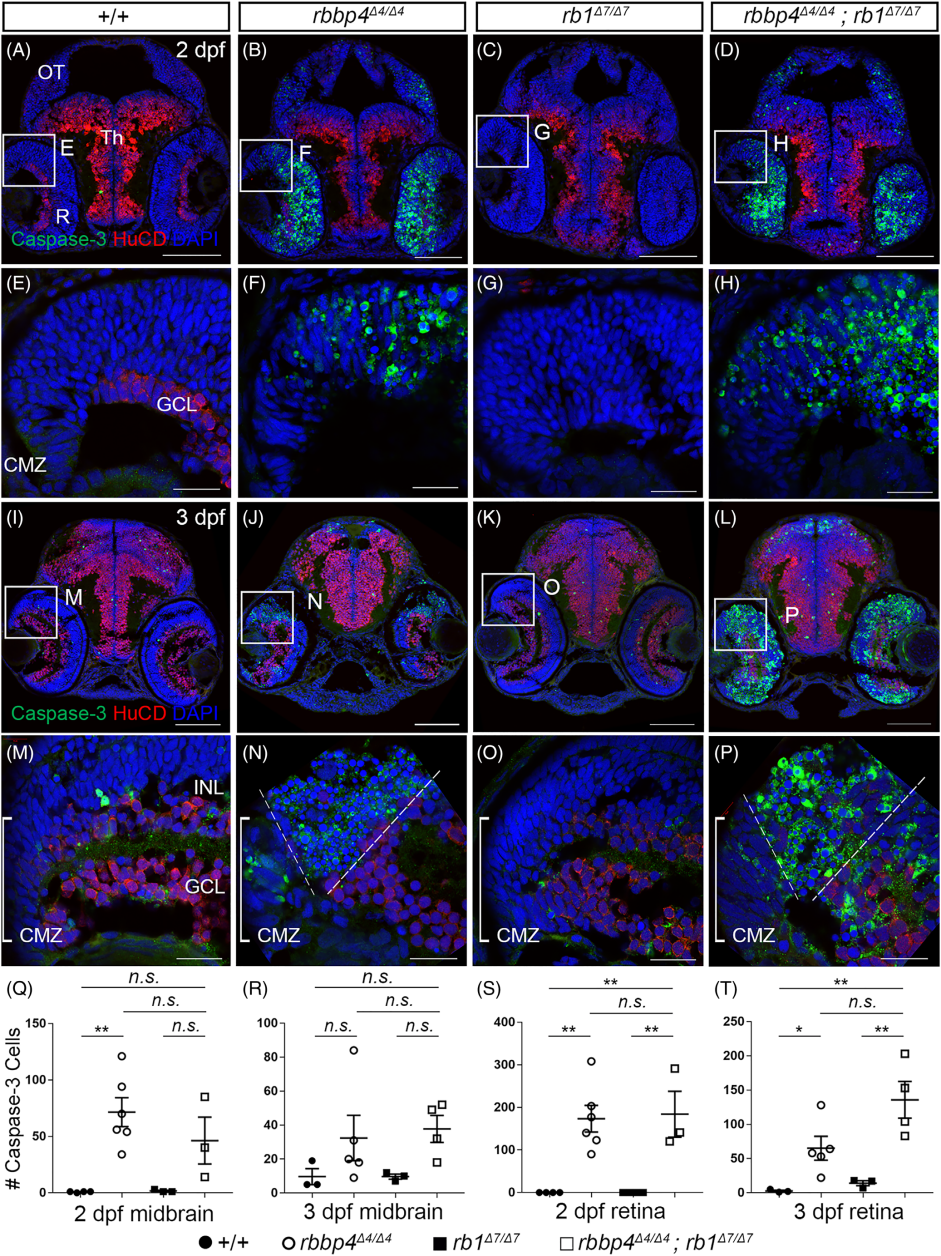
Rbbp4 is required for survival of *rb1* mutant retinal neural precursors. (A–H) 2 dpf and (I–P) 3 dpf wild type +/+ (n = 4, n = 3), *rbbp4* ^Δ4/Δ4^ (n = 6, n = 5), *rb1* ^Δ7/Δ7^ (n = 5, n = 4), and *rbbp4* ^Δ4/Δ4^; *rb1* ^Δ7/Δ7^ (n = 3, n = 2) siblings from a *rbbp4* ^Δ4/+^; *rb1* ^Δ7/+^ incross were sectioned and labeled with antibodies to activated Caspase‐3 and HuC/D. Boxed area in (A–D) and (I–L) is shown at higher magnification in (E–H) and (M–P), respectively. Dashed lines in (N) and (P) denote region adjacent to the ciliary marginal zone (brackets) where neural progenitors reside. (Q–T) Quantification of Caspase‐3 positive cells. *P* values correspond to comparisons moving left to right on plots. Statistical analysis was performed with one‐way ANOVA Sidak's multiple comparisons test. Plots show mean ± s.e.m. (Q) 2 dpf midbrain (**P = .0053, *P* = .1887 n.s., *P* = .6598 n.s., *P* = .2608 n.s.). (R) 3 dpf midbrain (*P* = .6420 n.s., *P* = .4520 n.s., *P* = .9993 n.s., *P* = .4520 n.s.). (S) 2 dpf retina (**P = .0038, **P = .0089, *P* > .9999 n.s., **P = .0089). (T) 3 dpf retina (*P = .0358, **P = .0035, *P* = .0859 n.s., **P = .0068). CMZ, ciliary marginal zone; GCL, ganglion cell layer; INL, inner nuclear layer; ONL, outer nuclear layer; OT, optic tectum; R, retina; Th, thalamic region. Scale bars: A–D, I–L 100 μm; E–H, M–P 20 μm

To determine whether survival of *rb1* mutant neural precursors is dependent on Rbbp4, we compared the levels of apoptosis in +/+, *rbbp4* ^Δ4/Δ4^, *rb1* ^Δ7/Δ7^ and *rbbp4* ^Δ4/Δ4^; *rb1* ^Δ7/Δ7^ double mutants at 2 dpf and 3 dpf (Figure 8A–P). In the midbrain only *rbbp4* ^Δ4/Δ4^ mutants at 2 dpf showed a significant elevation in activated Caspase‐3 labeling in comparison to wild type (*P* = .0053) (Figure 8Q,R). Apoptosis was not detected in the ciliary marginal zone of the retina at 2 or 3 dpf in any genotype (Figure 8E–H CMZ, M–P CMZ brackets) but could be detected in the inner nuclear layer and the progenitor region of the CMZ (Figure 8N,P dashed lines). The level of activated Caspase‐3 cells in *rbbp4* ^Δ4/Δ4^ single and *rbbp4* ^Δ4/Δ4^; *rb1* ^Δ7/Δ7^ double mutant retinas was not significantly different at 2 dpf (*P* > .9999) or 3 dpf (*P* = .0859). “These results show that loss of Rbbp4 can induce apoptosis in *rb1* mutant neural precursors, further supporting a role for Rbbp4 in neural progenitor survival that is independent of Rb.”

### 2.7 *tp53* morpholino knockdown suppresses *rbbp4* mutant neural precursor apoptosis and γ‐H2AX

**FIGURE 9:**
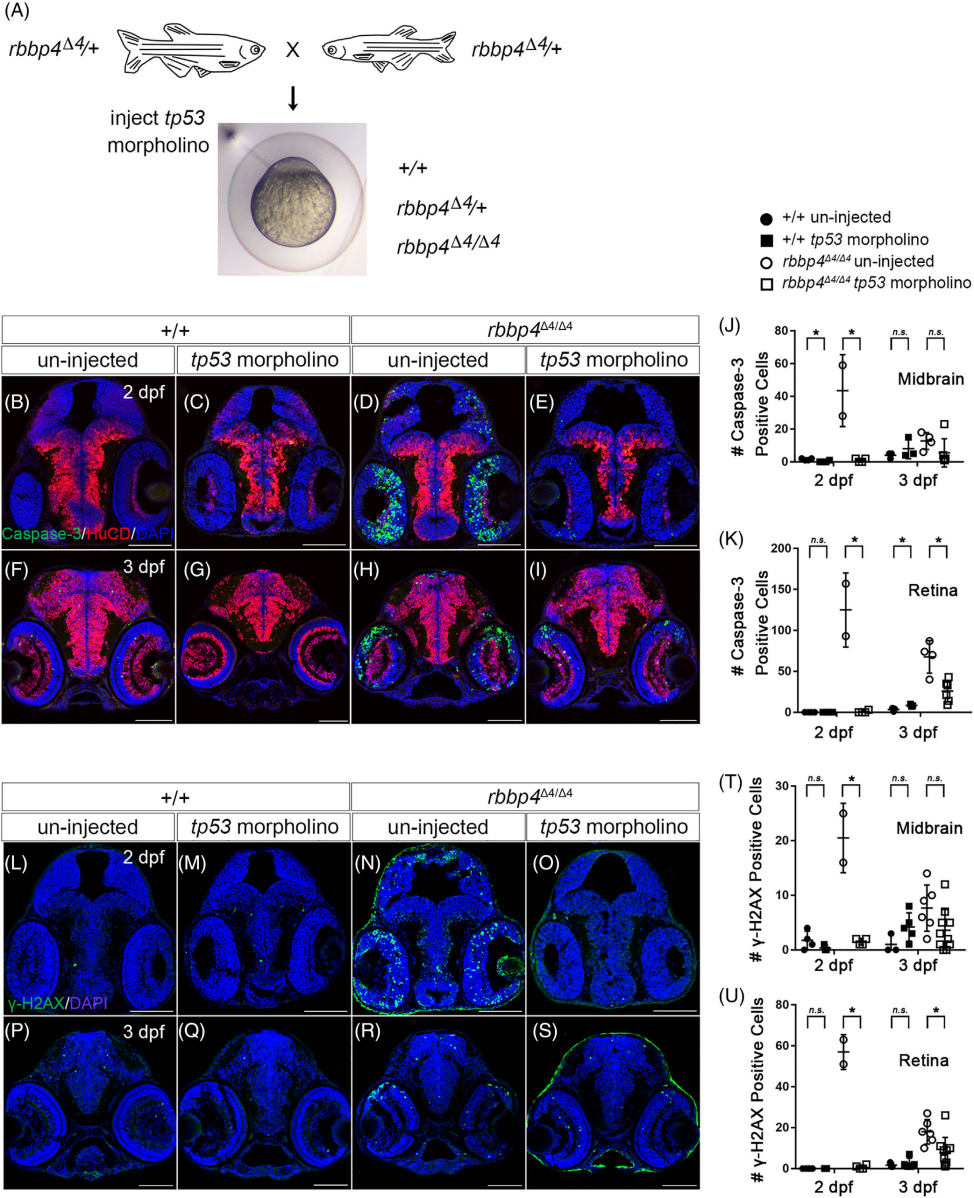
Morpholino knockdown of *tp53* suppresses apoptosis in *rbbp4* mutant neural precursors. (A) Inhibition of Tp53 activity by antisense morpholino injection into embryos from *rbbp4* ^Δ4^ */+* adult incross. Image of 1 cell embryo from Almeida et al.^32^ (B–U) 2 dpf and 3 dpf wild type and *rbbp4* ^Δ4/Δ4^ larval midbrain sections after *tp53* knockdown. Activated Caspase‐3 (green), HuC/D (red) and DAPI (blue) labeling at 2 dpf (B–E) and 3 dpf (F–I). (J, K) Quantification of Caspase‐3 positive cells in the midbrain and retina at 2 dpf (un‐injected +/+ n = 4; un‐injected *rbbp4* ^Δ4/Δ4^ n = 2; injected +/+ n = 4; injected *rbbp4* ^Δ4/Δ4^ n = 4) and 3 dpf (un‐injected +/+ n = 3; un‐injected *rbbp4* ^Δ4/Δ4^ n = 4; injected +/+ n = 3; injected *rbbp4* ^Δ4/Δ4^ n = 6). (J) 2 dpf midbrain +/+ un‐injected vs injected (*P* = .0170*), *rbbp4* ^Δ4/Δ4^ un‐injected vs injected (*P = .0112). The 3 dpf midbrain +/+ un‐injected vs injected (*P* = .3349 n.s.), *rbbp4* ^Δ4/Δ4^ un‐injected vs injected (*P* = .1736 n.s.). (K) 2 dpf retina +/+ un‐injected and injected quantification values were zero, *rbbp4* ^Δ4/Δ4^ un‐injected vs injected (*P = .0032). The 3 dpf +/+ un‐injected vs injected (*P = .0285), *rbbp4* ^Δ4/Δ4^ un‐injected vs injected (*P = .0037). (L–S) γ‐H2AX labeling (green), and nuclear stain DAPI (blue) at 2 dpf (L–O) and 3 dpf (P–S). (T) Comparison of γ‐H2AX positive cells in the midbrain between un‐injected and *tp53* MO injected wild type at 2 dpf (un‐injected n = 4; *tp53* MO injected n = 3; *P* = .2344), *rbbp4* ^Δ4/Δ4^ at 2 dpf (un‐injected n = 2; *tp53* MO injected n = 4; *P* = .0024), wildtype at 3 dpf (un‐injected n = 3; *tp53* MO injected n = 5; *P* = .1101), and *rbbp4* ^Δ4/Δ4^ at 3 dpf (un‐injected n = 6; *tp53* MO injected n = 10; *P* = .0657). (U) Comparison of γ‐H2AX positive cells in the retina between un‐injected and *tp53* MO injected wildtype at 2 dpf (un‐injected n = 4; *tp53* MO injected n = 3; *P*‐value = 0), *rbbp4* ^Δ4/Δ4^ at 2 dpf (un‐injected n = 2; *tp53* MO injected n = 4; *P* = .0001), wildtype at 3 dpf (un‐injected n = 3; *tp53* MO injected n = 5; *P* = .5784), and *rbbp4* ^Δ4/Δ4^ at 3 dpf (un‐injected n = 6; *tp53* MO injected n = 10; *P* = .0128). Statistical analysis was performed with one‐tailed Student's *t*‐test. Plots show mean ± s.e.m. Scale bars: 100 μm

To test whether apoptosis in *rbbp4* ^Δ4/Δ4^ mutants was dependent on Tp53, *tp53* expression was knocked down by injection of an antisense translation blocking *tp53* morpholino into 1‐cell stage embryos (Figure 9A). Control un‐injected and injected wild type and *rbbp4* ^Δ4/Δ4^ mutant embryos were sectioned at 2 and 3 dpf and double labeled with antibodies to activated Caspase‐3 and neuronal differentiation marker HuC/D (Figure 9B–I). Compared to wild‐type un‐injected control embryos, Tp53 knock down did not affect development or cell viability in the wild‐type midbrain or retina at 2 dpf (Figure 9B,C) and 3 dpf (Figure 9F,G). In comparison to un‐injected *rbbp4* ^Δ4/Δ4^ embryos, Tp53 knock down suppressed activated Caspase‐3 labeling and apoptosis in the *rbbp4* ^Δ4/Δ4^ midbrain and retina at 2 dpf (Figure 9D,E) and 3 dpf (Figure 9D,E). Suppression of apoptosis in the midbrain was significant at 2 dpf, but not at 3 dpf, possibly since the overall number of apoptotic cells in the *rbbp4* ^Δ4/Δ4^ mutant midbrain was reduced at this later stage (Figure 9J). In the retina, tp53 knockdown significantly reduced the number of activated Caspase‐3 cells at both 2 and 3 dpf (Figure 9K). Together these results demonstrate that the activation of programmed cell death in *rbbp4* ^Δ4/Δ4^ mutant neural progenitors is dependent on tp53 signaling.

γ‐H2AX is routinely used as a marker for DNA damage associated with induction of TP53‐dependent apoptosis but also shows extensive labeling throughout cells and degrading nuclei at late stages of apoptosis. We examined γ‐H2AX labeling in *rbbp4* mutant larvae with and without *tp53* morpholino knockdown in 2 dpf (Figure 9L–O) and 3 dpf larvae (Figure 9P–S). At 2 dpf intense γ‐H2AX labeling can be detected in the *rbbp4* ^Δ4/Δ4^ midbrain and retina (Figure 9N) where activated Caspase‐3 is present and becomes similarly restricted at 3 dpf to proliferative zones (Figure 9R). Morpholino knockdown of *tp53* nearly completely suppressed γ‐H2AX labeling in *rbbp4* ^Δ4/Δ4^ 2 and 3 dpf larvae (Figure 9O,S,T,U). The lack of γ‐H2AX labeling in *rbbp4* ^Δ4/Δ4^ mutants after suppression of apoptosis by *tp53* knockdown suggests that the γ‐H2AX signal in the mutants may not represent DNA damage, but the end stage of cells undergoing apoptosis. This indicates loss of Rbbp4 may activate TP53 through a mechanism or pathway distinct from the canonical DNA damage response.

### 2.8 Maternal zygotic *tp53*Δ^is55^ deletion suppresses *rbbp4* mutant neural precursor apoptosis

**FIGURE 10:**
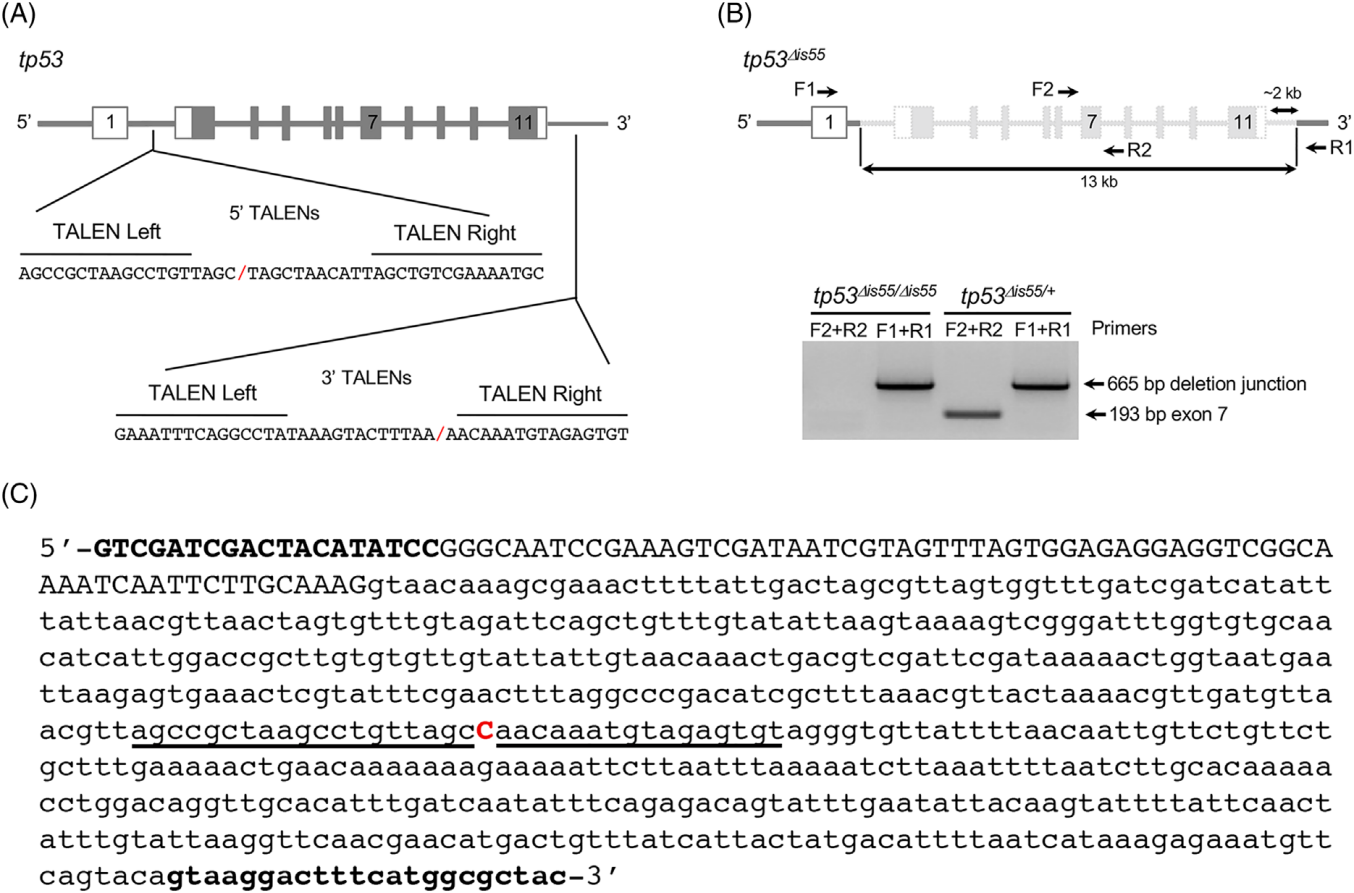
The zebrafish *tp53Δis55* deletion allele is homozygous viable. (A) Diagram of zebrafish *tp53* gene with TALEN pair recognition sequences targeting intron 1 (5′ TALEN pair) and ~2 kb downstream of the 3′ UTR (3′ TALEN pair). Red backslash represents location of 5′ and 3′ sides of the junction in the *tp53*Δ*is55* allele. (B) Diagram of zebrafish *tp53*Δ*is55* allele. ~13 kb of deleted genomic sequences are indicated in light grey. Arrows indicate location of primers F1 and R1 used to amplify the deletion allele junction, and primers F2 and R2 that flank exon 7 used to amplify exon 7 in the wild type *tp53* allele. Bottom gel shows PCR amplicon genotyping of homozygous *tp53*Δ*is*55/Δi*s*55 and heterozygous *tp53*Δ*is*55/+ adult zebrafish. (C) Sequence of F1/R1 PCR amplicon surrounding the 5′‐3′ junction between intron 1 and the 3′ UTR in the *tp53*Δ*is55* allele. 5′ TALEN Left and 3′ TALEN Right sequences are underlined. Red capital C is an insertion between the 5′ and 3′ sides of the junction allele. Sequences of forward (F1) and reverse (R1) primers are in bold. Uppercase letters represent exon 1

To demonstrate a genetic requirement for Tp53 in apoptosis after loss of Rbbp4, we used two TALEN pairs targeting sites near the 5′ and 3′ ends of the *tp53* gene to generate the deletion allele *tp53*Δ^is55^. The left TALEN pair target site was located in intron 1, and the right TALEN pair site was located in the 3′ UTR downstream of exon 11 (Figure 10A). The sequences of the TALENs (Table 2) were previously reported by Ignatius et al., in which a *tp53* ^*del*^ deletion allele was isolated; however, that allele was generated with a different 5′ TALEN pair that sits upstream of *tp53* exon 1.^33^ The *tp53*Δ^is55^ allele deletes ~13 kb and is homozygous viable (Figure 10B). Analysis of the deletion junction shows sequences upstream of the 5**′** TALEN Left cut site and downstream of the 3′ TALEN Right cut site fused together, with a single C inserted in between (Figure 10C).

**TABLE 2:** Sequences of oligonucleotides and TALENs used in this study

Oligonucleotides	Forward 5′‐3′	Reverse 5′‐3′	
*rbbp4* ^Δ4*is*60^ exon 2 allele genotyping primers	GCGTGATGACAGATCTCATATTGTTTTCCC	CTGGTGACATCTGGCAACCACT	Schultz et al.^17^
*rb1* ^Δ7*is*54^ exon 2 allele genotyping primers	TTTCCAGACACAAGGACAAGGATCC	GCAGATATCAGAAGAAAGAGTACATTTGTCTT	Solin et al.^16^
*rbbp4* cDNA	catgTCTAGATGTGGAGTCGTTATGGCTG	catgGGATCCTCCCTGAACCTCAGTGTCTG	This paper
*mz98* cDNA	CCGGACACTACACACTCAATGC	GTGCTGGATGTAGCTGTTCTCG	This paper
*ccnD1* cDNA	GCGAAGTGGATACCATAAGAAGAGC	GCTCTGATGTATAGGCAGTTTGG	This paper
*atoh7* cDNA	GATTCCAGAGACCCGGAGAAG	CAGAGGCTTTCGTAGTGGTAGGAG	This paper
*cdkn1c* cDNA	CGTGGACGTATCAAGCAATCTGG	GTCTGTAATTTGCGGCGTGC	This paper
*tp53* ^Δ*is*55^ F1 and R1 deletion allele genotyping primers	GTCGATCGACTACATATCCG	GTAGCGCCATGAAAGTCCTTA	This paper
*tp53* F2 and R2 exon 7 genotyping primers	GTCTGTCCATCTGTTTAACAGTC	AGCAGTTCACAAGAGGAGGAATC	This paper

TALENs	TALEN target nucleotide sequence 5′‐3′	TAL domain amino acid sequence	
*tp53* 5′ intron 1 TALEN left	AGCCGCTAAGCCTGT	NI NN HD NN HD NG NI NI NN HD HD NG NN NG	Ignatius et al.^33^
*tp53* 5′ intron 1 TALEN right	GCATTTTCGACAGCT	NN HD NI NG NG NG NG HD NN NI HD NI NN HD NG	Ignatius et al.^33^
*tp53* 3′ UTR TALEN left	GAAATTTCAGGCCTA	NN NI NI NI NG NG NG HD NI NN NN HD HD NG NI	Ignatius et al.^33^
*tp53* 3′ UTR TALEN right	ACACTCTACATTTGT	NI HD NI HD NG HD NG NI HD NI NG NG NG NN NG	Ignatius et al.^33^

*Note*: Oligonucleotide sequences of primers used for PCR genotyping and cDNA cloning to generate probes for in situ hybridization. The 4 TALEN recognition genomic target sites in *tp53* and corresponding amino acids of the TAL domain are listed.

**FIGURE 11:**
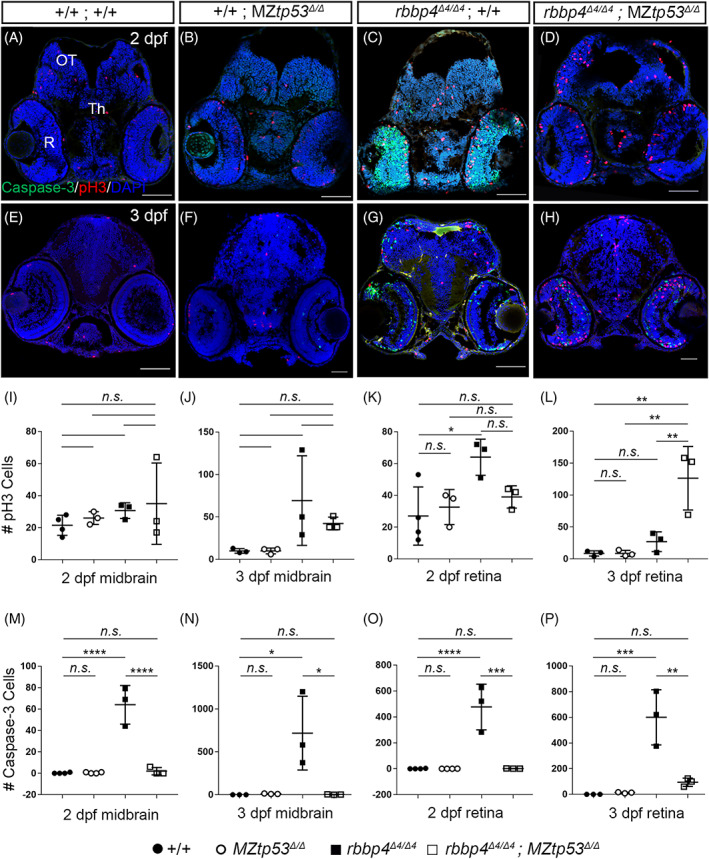
Maternal‐zygotic *tp53*Δ*is*55 deletion suppresses apoptosis in neural progenitors and reveals increased levels of mitotic cells in the *rbbp4* mutant retina. (A–D) 2 dpf and (E‐H) 3 dpf wild type +/+ (n = 4, n = 3), MZ*tp53* ^Δ/Δ^ (n = 4, n = 3), *rbbp4* ^Δ4/Δ4^ (n = 3, n = 3), *rbbp4* ^Δ4/Δ4^; MZ*tp53* ^Δ/Δ^ (n = 3, n = 3), siblings from a *rbbp4* ^Δ4/+^; MZ*tp53* ^Δ/Δ^ incross were sectioned and labeled with antibodies to activated Caspase‐3 and phosphohistone pH 3. (I–P) Quantification of pH 3 positive cells (I–L) and Caspase‐3 positive cells (M–P). *P* values correspond to comparisons starting at the highest line on each plot. Statistical analysis was performed with one‐way ANOVA Tukey's multiple comparisons test. Plots show mean ± s.e.m. (I) 2 dpf midbrain pH 3 cells (*P* = .5424 n.s., *P* = .8256 n.s., *P* = .9748 n.s., *P* = .7876 n.s., *P* = .9662 n.s.). (J) 3 dpf midbrain pH 3 cells (*P* = .4893 n.s., *P* = .4893 n.s., *P* = .6226 n.s., *P* = .0988 n.s., *P* > .9999 n.s.). (K) 2 dpf retina pH 3 cells (*P* = .6543 n.s., *P* = .9351 n.s., *P* = .1701 n.s., *P* = .0233*, *P* = .9425 n.s.). (L) 3 dpf retina pH 3 cells (**P = .0026, **P = .0026, **P = .0072**, *P* = .8267 n.s., *P* > .9999 n.s.). (M) 2 dpf midbrain Caspase‐3 cells (*P* = .9920 n.s., ****P < .0001, *P* > .9999 n.s., ****P < .0001). (N) 3 dpf midbrain Caspase‐3 cells (*P* > .9999 n.s., *P = .0147, *P* = .9999 n.s., *P = .0150). (O) 2 dpf retina Caspase‐3 cells (*P* > .9999 n.s., ****P < .0001 *P* > .9999 n.s., ***P = .0001). (P) 3 dpf retina Caspase‐3 cells (*P* = .7176 n.s., ***P = .0007, *P* = .9987 n.s., **P = .0020. CMZ, ciliary marginal zone; GCL, ganglion cell layer; INL, inner nuclear layer; ONL, outer nuclear layer; OT, optic tectum; R, retina; Th, thalamic region. Scale bars: A–D, I–L 100 μm; E–H, M–P 20 μm

The level of mitotic pH 3 and activated Caspase‐3 positive cells were analyzed in embryos from a *rbbp4* ^Δ4/+^; maternal zygotic (MZ) *tp53* ^Δis55/Δis55^ incross (Figure 11A–H). The number of pH 3 positive cells in the midbrain and retina at 2 dpf (Figure 11A–D) was not significantly different between single *rbbp4* ^Δ4/Δ4^ and double *rbbp4* ^Δ4/Δ4^; MZ*tp53* ^Δis55/Δis55^ mutants (Figure 11I–K). However, at 3 dpf, there was a significant increase in the number of pH 3 positive cells in the retina of *rbbp4* ^Δ4/Δ4^; MZ*tp53* ^Δis55/Δis55^ double mutants compared to either *rbbp4* ^Δ4/Δ4^ or MZ*tp53* ^Δis55/Δis55^ single mutants (Figure 11L). At 2 and 3 dpf, the level of activated Caspase‐3 in double *rbbp4* ^Δ4/Δ4^; MZ*tp53* ^Δis55/Δis55^ mutants was significantly reduced compared to single *rbbp4* ^Δ4/Δ4^ mutants but was not significantly different than wild type (Figure 11A,D,E,H,M–P). Together these results show that genetic deletion of *tp53* suppresses apoptosis to wild‐type levels in *rbbp4* ^Δ4/Δ4^ mutants. In addition, suppression of programmed cell death revealed an increased number of cells in the mitotic phase of the cell cycle in the *rbbp4* ^Δ4/Δ4^ mutant retina, suggesting a defect in cell cycle regulation and or progression.

### 2.9 Rbbp4 loss leads to Tp53 acetylation, while overexpression fails to rescue apoptosis after chemically induced DNA damage

**FIGURE 12:**
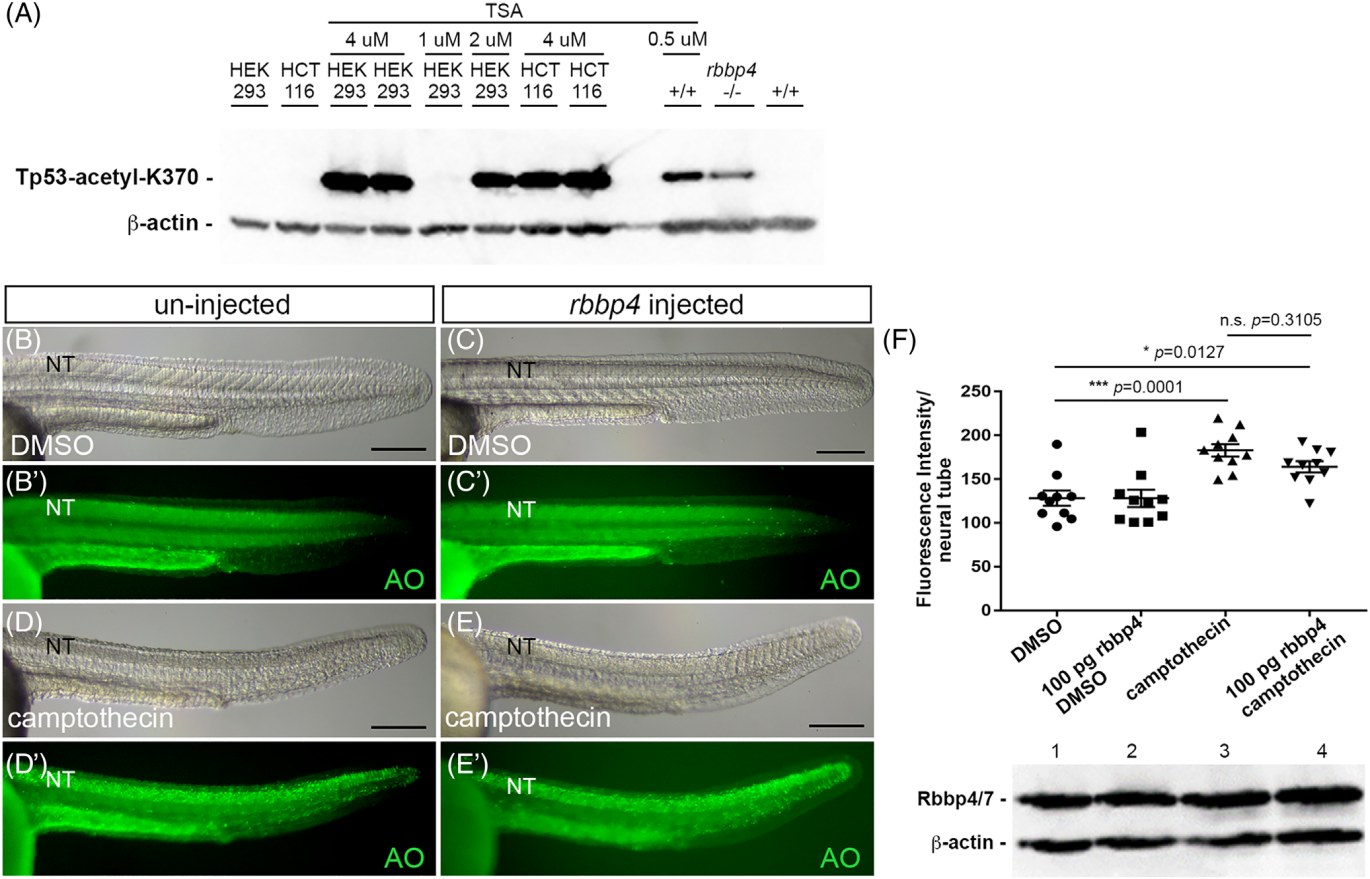
Loss of Rbbp4 leads to Tp53 acetylation but Rbbp4 overexpression does not rescue camptothecin induction of DNA damage and apoptosis. (A) Western blot probed with anti‐Tp53‐acetylated‐K370 and loading control anti‐β‐actin. Lane 1 HEK293 cells treated with DMSO; lane 2 HCT116 cells treated with DMSO; lane 3 HEK293 cells treated with 4 μM Tricostatin A (TSA) for 8 hours (hrs); lane 4 HEK293 cells treated with 4 μM TSA for 24 hours; lane 5 HEK293 cells treated with 1 uM TSA for 24 hours; lane 6 HEK293 cells treated with 2μM TSA for 24 hours; lane 7 HCT116 cells treated with 4 μM TSA for 8 hours; lane 8 HCT116 cells treated with 4 μM TSA for 24 hours; lane 9 underloaded sample; lane 10 wild‐type zebrafish WIK embryos treated with 0.5 μM TSA for 48 hours; 48 hpf *rbbp4* ^Δ4/Δ4^ homozygous mutant embryos, 48 hpf wildtype WIK embryos. (B–F) 30 hpf bright field and fluorescence images of the trunks of uninjected (B–B′), 100 pg *rbbp4* mRNA injected (C–C′), 100um camptothecin treated (D–D′), and 100 pg *rbbp4* mRNA injected plus 100 μm camptothecin treated (E–E') embryos labeled with acridine orange (AO). (F) Quantification of acridine orange fluorescence labeling in the neural tube (n = 10 embryos/condition). Western blot of protein extracted from 30 hpf zebrafish embryos probed with anti‐RBBP4/7 and loading control anti‐β‐actin. Lanes 1‐4:1. DMSO treated; 2. 100 μm camptothecin treated; 3. 100 pg *rbbp4* mRNA injected DMSO treated; 4. 100 pg *rbbp4* mRNA injected 100 μm camptothecin treated. Statistical analysis was performed with two‐way ANOVA with multiple comparisons test. Plots show mean ± s.e.m. NT, neural tube. Scale bars, 200 μm

To investigate the role of Rbbp4 in activation of Tp53‐dependent apoptosis, we examined how the loss of Rbbp4 impacts Tp53 acetylation. Tp53 transcriptional activity is known to be activated by p300‐mediated acetylation of C‐terminal lysine residues and repressed by histone deacetylase 1.^34^ Since Rbbp4 is a component of the histone deacetylase 1 NuRD complex, we examined whether the loss of Rbb4 leads to increased Tp53 acetylation at a lysine amino acid p300 target (Figure 12A). Western blotting with anti‐Tp53‐acetyl‐K370 antibody of human HEK293 cells, human HCT116 cells, and wild‐type +/+ 24 hpf zebrafish embryos treated with the histone deacetylase inhibitor Trichostatin A (TSA) show high levels of Tp53‐acetyl‐K370 in comparison to untreated cells and embryos. In contrast to wild type, Tp53‐acetyl‐K370 is present in homozygous mutant *rbbp4* ^Δ4/Δ4^ embryos. These results show that the loss of Rbbp4 leads to increased Tp53 acetylation.

The suppression of γ‐H2AX labeling in *rbbp4* ^Δ4/Δ4^ mutants after *tp53* knockdown suggested induction of apoptosis may occur through a pathway independent of the DNA damage response. To examine whether Rbbp4 functions in the DNA damage response pathway, we tested whether overexpression of Rbbp4 by mRNA injection would rescue DNA‐damage induced apoptosis. Wild‐type WIK embryos were injected with 100 pg of in vitro transcribed *rbbp4* mRNA. At 24 hpf, the embryos were treated with 100 μm camptothecin for 5 hours and labeled with acridine orange to visualize cells undergoing apoptosis (Figure 12B–E). Compared to control uninjected WIK embryos treated with camptothecin (Figure 12B–B',D–D'), injection of 100 pg *rbbp4* mRNA did not appear to reduce the amount of acridine orange labeling in the neural tube (Figure 12C–C', E–E'). Quantification of the level of acridine orange in the neural tube showed no significant difference between control camptothecin‐treated embryos and those injected with *rbbp4* mRNA (Figure 12F). A western blot of control, camptothecin treated, injected, and injected plus camptothecin‐treated embryos probed with an anti‐Rbbp4/7 antibody showed a slight elevation in the level of Rbbp4 protein after *rbbp4* mRNA injection. These results suggest that overexpression of Rbbp4 alone is not sufficient to rescue induction of apoptosis caused by DNA damage.

Together, the increase in Tp53 acetylation in *rbbp4* ^Δ4/Δ4^ mutants, and lack of rescue of apoptosis after Rbbp4 overexpression, suggest one mechanism driving apoptosis after loss of Rbbp4 is disruption of Rbbp4‐containing histone deacetylase complexes that regulate Tp53 acetylation and transcriptional activation.

## 3 DISCUSSION

In this study, we demonstrate that Rbbp4 is essential for zebrafish neurogenesis and its loss leads to Tp53‐dependent programmed cell death in neural precursors. Extensive apoptosis and elevated numbers of M‐phase cells are observed in the *rbbp4* mutant midbrain and retina. Loss of Rbbp4 and Rb together led to an additive increase in cells in M‐phase, suggesting that Rbbp4 has a role in cell cycle regulation independent of Rb. *rbbp4*; *rb1* mutant neural precursors undergo apoptosis, further supporting Rbbp4 functioning independently of Rb, and demonstrating Rbbp4 is required for survival of neural progenitors after loss of RB. We show evidence that the loss of Rbbp4 leads to Tp53 acetylation that correlates with induction of apoptosis. These results indicate multiple roles for Rbbp4 in regulating neural progenitor cell cycle progression and survival. Rbbp4 is overexpressed in zebrafish *rb1*‐embryonal brain tumors^17^; and in this study, we show human *RBBP4* is upregulated across the spectrum of human malignant embryonal and glial tumor types. Together with recent studies examining the role of human RBBP4 in glioblastoma DNA damage repair^18^ and neuroblastoma tumor progression,^19^ our study suggests that blocking Rbbp4 activity could prevent cell survival in Rb‐deficient brain cancer through inhibition of Tp53 deacetylation.

Our analysis of zebrafish *rbbp4* mutants revealed Rbbp4 is necessary for cell survival as well as cell cycle control during neurogenesis in the developing midbrain and retina. Apoptosis of midbrain neural progenitors could be detected as early as 36 hpf in the *rbbp4* mutant optic tectum, but the number of mitotic M phase cells was not significantly different than wild type. By 2 dpf, the number of apoptotic cells and M‐phase cells had increased significantly in the midbrain and retina. One interpretation of this observation is that it reflects the requirement for Rbbp4 in regulation of cell cycle entry as a component of the NuRD complex, which cooperates with the Rb tumor suppressor to block expression of E2F‐regulated early S phase genes.^9^ However, in *rbbp4*; *rb1* double mutants, the number of cells in M phase was enhanced but additive, indicating independent roles for Rb and Rbbp4 in cell cycle regulation. These differences may reflect vertebrate or neural specific requirements for Rbbp4 in progenitor cell cycle progression. Examining the requirement for Rbbp4 in cell cycle gene expression will lend new insight into the mechanism by which Rbbp4 regulates the neural progenitor cell cycle.

The observation that the loss of Rbbp4 leads to accumulation of cells in M‐phase is more consistent with a role for Rbbp4 in regulating cell cycle exit and differentiation. Rbbp4 is a component of the MuvB complex which regulates multiple stages of cell cycle gene expression.^12^ MuvB is part of the larger DREAM complex which represses cell cycle gene expression in G0/G1.^35^ MuvB associates with B‐Myb to form the MBB complex and activate gene expression necessary for progression through S/G2.^36^ At the G2/M transition, MuvB loses association with B‐Myb and forms a complex with the transcription factor FoxM1 to drive transcriptional activation of late cell cycle genes.^36^ Our analysis of pH 3 labeling does not determine whether Rbbp4 is required for MBB regulation of S/G2 progression, but it is consistent with a role for Rbbp4 in the MuvB/FoxM1 complex that drives progression through M phase. This interpretation is supported by the absence of expression of the G1 to G0 transition cell cycle inhibitor *cdkn1c*.

Our study demonstrates that the loss of Rbbp4 leads to Tp53‐dependent apoptosis in zebrafish neural progenitors and Tp53 acetylation. Tp53‐mediated transcriptional activation in response to DNA damage is stimulated by p300/CBP and PCAF acetylation.^37^, ^38^, ^39^ Studies in cell culture show Tp53 induces cell cycle arrest and apoptosis by targeted expression of the G1 cyclin dependent kinase inhibitor *cdkn1a/p21*.^22^ Tp53‐mediated cell cycle arrest and apoptosis are inhibited by deacetylation of Tp53 by the NuRD/HDAC1 complex.^22^ In cancer, NuRD‐mediated deacetylation is thought to repress Tp53 cell cycle arrest and apoptosis and to stabilize HIF1a and gene expression promoting metastasis.^40^ The observation that loss of Rbbp4 stimulates Tp53‐dependent apoptosis and Tp53 acetylation suggests a defect in NuRD‐mediated Tp53 deacetylation, which would increase acetylated‐Tp53 and stimulate Tp53‐dependent apoptosis. Preventing Tp53 activation through NuRD may provide a mechanism by which Rbbp4 promotes neural progenitor proliferation and contributes to brain tumor oncogenesis.

In the *rbbp4* mutant retina, the expansion of the *atoh7*‐positive progenitor population and the absence of *cdkn1c*‐expressing cells suggest that progenitors fail to transition to a differentiated state and initiate G0. The elevated level of M‐phase cells in *rbbp4* and *rbbp4*; maternal zygotic *tp53* double mutants suggests the G1/G0 transition defect is due to a failure of progenitors to exit the cell cycle. Loss of Rbbp4 may lead to altered transcriptional regulation of late cell cycle genes by the FoxM1/MuvB complex, leading to arrest in M phase. Alternatively, loss of Rbbp4 could affect PRC2‐mediated cell cycle gene silencing and heterochromatin formation required for cell cycle exit and differentiation.^41^ In Arabidopsis, the Rbbp4 homolog *AtMSI1* is necessary for persistent growth of reproductive tissues through expression of homeotic genes and heterochromatin assembly.^42^ Knockdown of Rbbp4 in cultured chicken DT40 cells leads to defects in DNA synthesis, nucleosome assembly, heterochromatin formation, and accumulation of cells in G2/M leading to cell death.^43^ Our results are consistent with these observations that Rbbp4 is required for cell cycle exit and terminal differentiation. The ability to suppress apoptosis by Tp53 knockdown or genetic deletion in zebrafish *rbbp4* mutants provides an opportunity to examine in vivo how Rbbp4 loss impacts cell cycle gene expression and heterochromatin formation required for progression of neural precursors to quiescence.

BrdU lineage tracing showed loss of Rbbp4 lead to cell death in the inner part of the retina but did not affect the proliferation or survival of retinal stem cells located at the periphery of the ciliary marginal zone. This observation contrasts with the requirement for Rbbp4 to maintain embryonic stem cell pluripotency,^44^ which suggests that after early embryogenesis Rbbp4 may no longer be required in tissue specific stem cell populations later in development. Conditional inactivation of Rbbp4 in retinal neural stem and progenitor populations in vivo would address whether Rbbp4 has a role in neural stem cell maintenance that is distinct from its function in neural progenitor cell cycle regulation and survival.

In summary, our study indicates that Rbbp4 may promote proliferation and survival of neural precursors by driving cell cycle progression independent of Rb and preventing activation of Tp53‐dependent apoptosis. While these results do not rule out the possibility that the loss of Rbbp4 leads to Tp53 activation through the DNA damage response pathway, they suggest one of the mechanisms driving apoptosis is disruption of chromatin remodeling complexes that directly regulate Tp53 acetylation and transcriptional activity. Together, our analysis provides the foundation for future studies examining the mechanisms by which Rbbp4 and its associated chromatin remodelers promote cell cycle progression and survival during neurogenesis.

## 4 EXPERIMENTAL PROCEDURES

### 4.1 Zebrafish care and husbandry

Zebrafish were reared in an Aquatic Habitat system (Aquatic Ecosystems, Inc., Apopka, FL). Fish were maintained on a 14‐hours light/dark cycle at 27°C. Transgenic lines were established in a WIK wild‐type strain obtained from the Zebrafish International Research Center (http://zebrafish.org/zirc/home/guide.php). The zebrafish *rbbp4* and *rb1* alleles used in this study were isolated by CRISPR‐Cas9 or TALEN targeting and described previously: *rbbp4* 4 base pair (bp) deletion allele *rbbp4* ^Δ4is60^ ^17^; *rb1* 7 bp deletion allele *rb1* ^Δ7is54^.^16^ For in situ hybridization and immunohistochemistry experiments, embryos were collected and maintained at 28.5°C in embryo media^45^ until harvesting. Embryos were staged according to published guidelines.^46^ All experimental protocols were approved by the Iowa State University Institutional Animal Care and Use Committee (IACUC‐18‐379, IBC‐18‐177) in compliance with American Veterinary Medical Association and the National Institutes of Health guidelines for the humane use of laboratory animals in research.

### 4.2 Larval genotyping and *tp53* morpholino injections

Primers to amplify *rbbp4* exon 2 containing the *rbbp4* ^Δ4is60^ mutation: Forward 5′ GCGTGATGACAGATCTCATATTGTTTTCCC 3′; Reverse 5′ CTGGTGACATCTGGCAACCACT 3′. Primers to amplify *rb1* exon 2 containing the *rb1* ^Δ7is54^ mutation: forward 5'‐TTTCCAGACACAAGGACAAGGATCC‐3′; reverse 5′‐GCAGATATCAGAAGAAAGAGTACATTTGTCTT‐3′. To inhibit Tp53‐dependent apoptosis, embryos were injected at the one cell stage with 2 ng of *tp53* translation blocking morpholino GCGCCATTGCTTTGCAAGAATTG^47^ (Gene Tools; ZDB‐MRPHLNO‐070126‐7).

### 4.3 Isolation of deletion allele *tp53* ^Δ*is*55^ and transgenic line *Tg(Tol2<ubi:rbbp4‐2AGFP>*)^*is*61^

The previously described TALEN pairs for targeted deletion of *tp53* (Table 2) were designed to a site in intron 1 and a site 2 kb downstream of exon 11.^33^ pT3TS‐TALEN vectors were linearized using *Sac*I and mRNA was in vitro transcribed using the mMessage mMachine T3 kit (ThermoFisher AM1348). The mRNA was purified using Qiagen RNeasy MinElute Cleanup Kit (Qiagen 74 204). A 40 pg of each TALEN mRNA was co‐injected into 1‐cell WIK embryos. Ten pairs of adults were screened for transmission of the 13 kb deletion allele by PCR of embryo genomic DNA with primers that span the deletion junction (Table 2). A single founder was identified and the deletion allele junction fragment confirmed by sequencing. A single heterozygous F1 adult was outcrossed to WIK to generate F2s, and a single heterozygous F2 was outcrossed to WIK to establish an F3 family of the *tp53* ^Δ*is*55^/+ line.

The *rbbp4* 1275 bp cDNA minus the translation termination codon was amplified by reverse transcription PCR with forward 5′‐catgTCTAGATGTGGAGTCGTTATGGCTG‐3′ and reverse 5′‐catgGGATCCTCCCTGAACCTCAGTGTCTG‐3′ primers that included 5′ *Xba*I and 3′ *BamH*I sites, respectively. The *Tol2<ubi:rbbp4‐2AGFP>* transgene was assembled using the NEBuilder HiFi DNA Assembly protocol and mix (New England Biolabs E2621S) in the mini‐*pTol2* vector.^48^ The zebrafish *ubiquitin* promoter^49^ was cloned into the vector followed by the *rbbp4* cDNA, an in‐frame 2A viral peptide GFP cassette^50^ and the zebrafish β‐*actin* 3'UTR (Dr. Darius Balciunas, Temple University^51^). A 1 μg of linearized *pT3TS‐Tol2* transposase plasmid^48^ was used as template for in vitro synthesis of *Tol2* transposase capped, polyadenylated mRNA with T3 mMessage mMachine High Yield Capped RNA transcription kit (ThermoFisher AM1348). Synthesized mRNA was precipitated with LiCl and resuspended in RNase, DNase‐free molecular grade water. The *Tg*(*Tol2<ubi:rbbp4‐2AGFP>*)^*i*s61^ transgenic line was isolated by co‐injection into 1‐cell WIK zebrafish embryos of 25 pg *Tol2* vector plus 100 pg *Tol2* mRNA. Fourteen founder fish were screened for germline transmission of ubiquitously expressed GFP. A single F1 adult that showed Mendelian transmission of the *Tol2<ubi:rbbp4‐2AGFP>* transgene to the F2 generation was used to establish the line.

### 4.4 Embryo *rbbp4* mRNA injections and camptothecin treatment

The *rbbp4* cDNA was cloned into the expression vector pT3TS. A 1 μg linearized vector was purified with the PureYield Plasmid Miniprep System (Promega, A1223) and used as template for in vitro synthesis of capped mRNA with the Ambion mMessage Machine T3 Transcription Kit (Thermo Fisher, AM1348). In vitro synthesized mRNA was purified with the RNA Clean and Concentrator Kit RCC (Zymo, R1013). Single‐cell zebrafish embryos were injected with 100 pg or 300 pg *rbbp4* mRNA. At 24 hpf, the embryos were incubated for 5 hours with 100 μm Camptothecin (Sigma‐Aldrich, C9911) or DMSO as a control. Embryos were incubated in 10ug/ml acridine orange (Thermo Fisher Scientific, AC423340010) in embryo media for 30 minutes. Acridine orange‐labeled living embryos were rinsed with embryo media and immediately imaged on a Zeiss Discovery.V12 stereomicroscope using a Cannon Rebel digital camera and EOS Utility software.

### 4.5 Immunohistochemistry

Embryonic (2 dpf) and larval (3 dpf) zebrafish were anesthetized in MS‐222 Tricaine Methanesulfonate and head and trunk dissected. Trunk tissue was placed in 20 μL 50 mM NaOH for genotyping. Heads were fixed in 4% paraformaldehyde overnight at 4°C, incubated in 30% sucrose overnight at 4°C, then processed and embedded in Tissue‐Tek OCT (Fisher 4583). Tissues were sectioned at 14–16 μm on a Microm HM 550 cryostat. For BrdU labeling experiments, 2 dpf embryos were incubated in 5 μM BrdU (Sigma B5002) in embryo media^45^ for 2.5 hours, placed in fresh fish water, then sacrificed immediately or at 3 dpf and 5 dpf. To aid BrdU antigen, retrieval tissues were pretreated with 2 M HCl. Antibodies used in this study: rabbit polyclonal anti‐phospho‐Histone H3 PH3 1:1000 (Cell Signaling Technology; 9701); mouse monoclonal anti‐phospho‐Histone H3 (Ser10), clone 3H10 1:500 (Millipore 05‐806); mouse monoclonal anti‐HuC/D 1:500 (Invitrogen A‐21271); rabbit polyclonal anti‐activated Caspase‐3 1:500 (BD Biosciences 559 565); mouse monoclonal anti‐BrdU 1∶500 (Bio‐Rad MCA2483); Alexa‐594 (Invitrogen A‐11005) and Alexa‐488 (Invitrogen A‐11008) conjugated secondary antibodies 1:500. Tissues were counterstained with 5 μg/mL DAPI, mounted in Fluoro‐Gel II containing DAPI (Electron Microscopy Sciences 17985‐50) and imaged on a Zeiss LSM700 laser scanning confocal.

### 4.6 Cell culture, histone deacetylase inhibitor treatment and Western Blotting

Human HEK293 and HCT116 cells were cultured in EMEM (ATCC, 30‐2003) and DMEM Gibco # 11995‐065) medium, respectively. Cells were treated with 4 μM histone deacetylase inhibitor Trichostatin A (TSA) (Sigma, T8552) or DMSO for 24 hours, then collected and lysed in NP40 buffer (Fisher, FNN0021) for Western blot analysis. Zebrafish wild‐type 24 hpf embryos were treated with 0.5 μM TSA for 24 and 48 hours. A 20 μg of total protein extracted from cell and zebrafish embryo samples was run on a 10% SDS‐PAGE gel, blotted to PVDF membrane (Bio Rad, 1620176), and incubated with rabbit monoclonal anti‐TP53‐acetylated‐K370 (abcam, ab183544) at 1:500, rabbit polyclonal anti‐β actin (Cell Signaling, 4967) at 1:2000, rabbit polyclonal anti‐RBBP4 (Bethyl A301‐206A‐T, RRID: AB_890631) 1:2000, and HRP‐conjugated anti‐rabbit secondary antibody (Invitrogen, 31 460) at 1:5000. Blots were developed with SuperSignal West Dura Extended Duration Substrate (ThermoScientific, 34075) and imaged on a ThermoFisher iBright 1500 system.

### 4.7 In situ hybridization and alcian blue staining

cDNA was amplified by reverse transcription‐polymerase chain reaction out of total RNA isolated from wild‐type 5 dpf embryos and cloned into the pCR4‐TOPO vector (Invitrogen). Primers for amplification: *mz98* forward 5′‐CCGGACACTACACACTCAATGC‐3′, *mz98* reverse 5′‐GTGCTGGATGTAGCTGTTCTCG‐3′; *ccnD1* forward 5′‐GCGAAGTGGATACCATAAGAAGAGC‐3′, *ccnD*1 reverse 5′‐GCTCTGATGTATAGGCAGTTTGG‐3′; *atoh7* forward 5′‐GATTCCAGAGACCCGGAGAAG‐3′, *atoh7* reverse 5′‐CAGAGGCTTTCGTAGTGGTAGGAG‐3′; *cdkn1c* forward 5′‐CGTGGACGTATCAAGCAATCTGG‐3′, *cdkn1c* reverse 5′‐GTCTGTAATTTGCGGCGTGC‐3′. Digoxigenin‐labeled probes were synthesized using T3 RNA polymerase (Roche #11031163001) and DIG RNA labeling mix (Roche #11277073910) according to the manufacturer's instructions and stored in 50% formamide at −20°C. Embryonic and larval zebrafish tissues were fixed in 4% paraformaldehyde and embedded in Tissue‐Tek OCT (Fisher 4583). In situ hybridization was performed on 14–16 μm cryosections. For alcian blue staining of cartilage, zebrafish larvae were anesthetized and fixed in 4% paraformaldehyde overnight at 4°C and incubated in 0.1% alcian blue solution overnight at 4°C. Embryos were rinsed in acidic ethanol and stored in 70% glycerol. Whole larvae and tissue sections were imaged on a Zeiss Discovery.V12 stereomicroscope or Zeiss Axioskop 2 microscope and photographed with a Cannon Rebel camera.

## 5 QUANTIFICATION AND STATISTICAL ANALYSIS

Statistical analyses using unpaired, two tailed Student's *t*‐test; unpaired, two tailed Welch's *t*‐test; one‐way ANOVA Sidak's multiple comparisons test; and two‐ANOVA multiple comparison test, were performed using GraphPad Prism software. Statistical parameters are included in the figure legends. Data plots represent mean ± s.e.m.

## AUTHOR CONTRIBUTIONS

**Laura E. Schultz‐Rogers:** Conceptualization (lead); formal analysis (lead); investigation (lead); methodology (lead); validation (lead); visualization (lead); writing – original draft (lead); writing – review and editing (lead). **Michelle L. Thayer:** Conceptualization (supporting); formal analysis (supporting); investigation (supporting); validation (supporting); visualization (supporting); writing – original draft (supporting); writing – review and editing (supporting). **Sekhar Kambakam:** Conceptualization (supporting); formal analysis (supporting); investigation (supporting); validation (supporting); visualization (supporting). **Wesley A. Wierson:** Conceptualization (supporting); formal analysis (supporting); investigation (supporting); methodology (supporting); validation (supporting). **Jordan A. Helmer:** Investigation (supporting); validation (supporting). **Mark D. Wishman:** Investigation (supporting); validation (supporting). **Kristen A. Wall:** Investigation (supporting); validation (supporting). **Jessica L. Greig:** Investigation (supporting); validation (supporting). **Jaimie L. Forsman:** Investigation (supporting); validation (supporting). **Kavya Puchhalapalli:** Investigation (supporting); validation (supporting). **Siddharth U. Nair:** Investigation (supporting); validation (supporting). **Trevor J. Weiss:** Investigation (supporting); methodology (supporting); validation (supporting). **Jon M. Luiken:** Investigation (supporting); validation (supporting). **Patrick R. Balckburn:** Conceptualization (supporting); investigation (supporting); methodology (supporting); validation (supporting). **Stephen C. Ekker:** Conceptualization (supporting); funding acquisition (lead); methodology (supporting); resources (supporting); supervision (supporting). **Marcel Kool:** Data curation (supporting); formal analysis (supporting); resources (supporting); software (supporting); visualization (supporting). **Maura McGrail:** Conceptualization (lead); formal analysis (lead); funding acquisition (lead); investigation (lead); methodology (lead); project administration (lead); resources (lead); supervision (lead); visualization (supporting); writing – original draft (lead); writing – review and editing (lead).
